# The *Caenorhabditis elegans* Homolog of Gen1/Yen1 Resolvases Links DNA Damage Signaling to DNA Double-Strand Break Repair

**DOI:** 10.1371/journal.pgen.1001025

**Published:** 2010-07-15

**Authors:** Aymeric P. Bailly, Alasdair Freeman, Julie Hall, Anne-Cécile Déclais, Arno Alpi, David M. J. Lilley, Shawn Ahmed, Anton Gartner

**Affiliations:** 1 Wellcome Trust Centre for Gene Regulation and Expression, University of Dundee, Dundee, United Kingdom; 2 Cancer Research United Kingdom Nucleic Acid Structure Research Group, University of Dundee, Dundee, United Kingdom; 3 Department of Genetics and Department of Biology, University of North Carolina, Chapel Hill, North Carolina, United States of America; Washington University, United States of America

## Abstract

DNA double-strand breaks (DSBs) can be repaired by homologous recombination (HR), which can involve Holliday junction (HJ) intermediates that are ultimately resolved by nucleolytic enzymes. An N-terminal fragment of human GEN1 has recently been shown to act as a Holliday junction resolvase, but little is known about the role of GEN-1 in vivo. Holliday junction resolution signifies the completion of DNA repair, a step that may be coupled to signaling proteins that regulate cell cycle progression in response to DNA damage. Using forward genetic approaches, we identified a *Caenorhabditis elegans* dual function DNA double-strand break repair and DNA damage signaling protein orthologous to the human GEN1 Holliday junction resolving enzyme. GEN-1 has biochemical activities related to the human enzyme and facilitates repair of DNA double-strand breaks, but is not essential for DNA double-strand break repair during meiotic recombination. Mutational analysis reveals that the DNA damage-signaling function of GEN-1 is separable from its role in DNA repair. GEN-1 promotes germ cell cycle arrest and apoptosis via a pathway that acts in parallel to the canonical DNA damage response pathway mediated by RPA loading, CHK1 activation, and CEP-1/p53–mediated apoptosis induction. Furthermore, GEN-1 acts redundantly with the 9-1-1 complex to ensure genome stability. Our study suggests that GEN-1 might act as a dual function Holliday junction resolvase that may coordinate DNA damage signaling with a late step in DNA double-strand break repair.

## Author Summary

Coordination of DNA repair with cell cycle progression and apoptosis is a central task of the DNA damage response machinery. A key intermediate of recombinational repair and meiotic recombination, first proposed in 1964, involves four-stranded DNA structures. These intermediates have to be resolved upon completion of DNA repair to allow for proper chromosome segregation. Using forward genetics, we identified a *Caenorhabditis elegans* dual function DNA double-strand break repair and DNA damage signaling protein orthologous to the human GEN1 Holliday junction resolving enzyme. GEN-1 facilitates repair of DNA double-strand breaks, but is not essential for DNA double-strand break repair during meiotic recombination. The DNA damage signaling function of GEN-1 is separable from its role in DNA repair. Unexpectedly, GEN-1 defines a DNA damage-signaling pathway that acts in parallel to the canonical pathway mediated by CHK-1 phosphorylation and CEP-1/p53. Thus, an enzyme that can resolve Holliday junctions may directly couple a late step in DNA repair to a pathway that regulates cell cycle progression in response to DNA damage.

## Introduction

The correct maintenance and duplication of genetic information is constantly challenged by genotoxic stress. DNA double-strand breaks (DSBs) are amongst the most deleterious lesions. DSBs can be induced by ionizing irradiation (IR) or caused by the stalling of DNA replication forks. In response to DSBs, cells activate conserved DNA damage checkpoint pathways that lead to DNA repair, to a transient cell cycle arrest, or to apoptosis and senescence. The full activation of DNA damage response pathways and DSB repair by homologous recombination (HR) depends on a series of nucleolytic processing events. Following DSB formation, broken ends are resected in a 5′ to 3′ direction to generate 3′ single-strand overhangs [1]. These tails are coated by RPA1 molecules, which in turn are thought to lead to the recruitment of the ATR checkpoint kinase [2]. This kinase, and the related kinase ATM, appear to be directly targeted to DNA double-strand breaks to act at the apex of the DNA damage signaling cascade [3]. The DNA damage specific clamp loader comprised of Rad17 bound to the four smallest RFC subunits [4] recruits a PCNA-like complex referred to as “9-1-1” complex to the dsDNA–ssDNA transition of resected DNA ends [5]–[7]. The 9-1-1 complex is needed for full ATR activation [8],[9]. DSB repair by HR proceeds by replacing RPA1 with the RAD51 recombinase [10], [11]. The resulting nucleoprotein filament invades an intact donor DNA to form a D-loop structure. The invading strand is extended using the intact donor strand as a template. Annealing of the 3′ single-stranded tail of the second resected DNA end to the displaced donor DNA strand (second end capture), and DNA ligation lead to the formation of a double Holliday junction (dHJ) intermediate (for a review, see [12]). This dHJ must be resolved either through cleavage by Holliday junction (HJ)-resolving enzymes or through “dissolution” by the combined activity of the Blooms helicase and topoisomerase III [13], [14].

Prototypic HJ resolving enzymes are nucleases that resolve HJs by introducing two symmetrical cleavages that result in either crossover or non-crossover products, depending on which strands are cleaved. Cuts made by junction-resolving enzymes need to be perfectly symmetrical so that products can be re-ligated, thus requiring no further processing events for HJ resolution [15], [16]. Until recently, the molecular nature of canonical HJ resolvases in animals and plants remained enigmatic despite the observation of HJ-resolving activity in cellular extracts over many years [17], [18]. Resolving enzymes have been purified from bacteriophages, bacteria and archea but the only eukaryotic resolving enzymes that had been discovered until recently were *S. cerevisiae* Cce1 and *S. pombe* Ydc2, both of which act in mitochondria [15], [19], [20]. One possible pathway of HJ resolution involves the conserved MUS81/EME1 complex, probably the principal meiotic resolution activity in fission yeast [21], [22], although mouse as well as budding yeast strains lacking Mus81 only have very minor meiotic phenotypes [23], [24]. By comparison with known resolving enzymes, the *in vitro* properties of this complex currently appear somewhat imprecise, and more akin to flap endonuclease action [25], yet recent evidence suggests that this complex can lead to productive HJ resolution [26], [27]. In addition, it was recently shown that a complex between the SLX4 scaffold protein and the SLX1 nuclease can act as an HJ resolving enzyme [28]–[30]. Intriguingly, SLX4 also interacts with the XPF and MUS81 nucleases, providing a scaffold for repairing multiple DNA structures and the sequential action of SLX4/nuclease complexes on HJ might rather be described as HJ processing that nevertheless ultimately leads to HJ resolution [28]–[32]. While recent studies suggest that the SLX4 scaffold and associated nucleases may promote nuclease-dependent HJ resolution, an independent enzyme with HJ resolution activity, mammalian GEN1, was identified *in vitro* via biochemical fractionation [33]. GEN1 generates symmetrical cleavage in a manner similar to the *E. coli* RuvC junction-resolving enzyme. In parallel the budding yeast GEN1 ortholog Yen1 was identified as a resolving enzyme using functional genomics based approaches. The biological functions of human GEN1 are unclear, and the deletion of *yen1* has no obvious DNA repair defect [34]. Furthermore, it is not clear how or even if the processing of HJs is coordinated with DNA damage signaling. Recent evidence suggests that deleting the budding yeast *yen1* in conjunction with *mus81* leads to MMS hyper-sensitivity [34]. Also, expressing human GEN1 in fission yeast, which does not encode for a *gen-1/yen1* homolog, complements the meiotic defect associated with mus81 [35].

**Figure 1:**
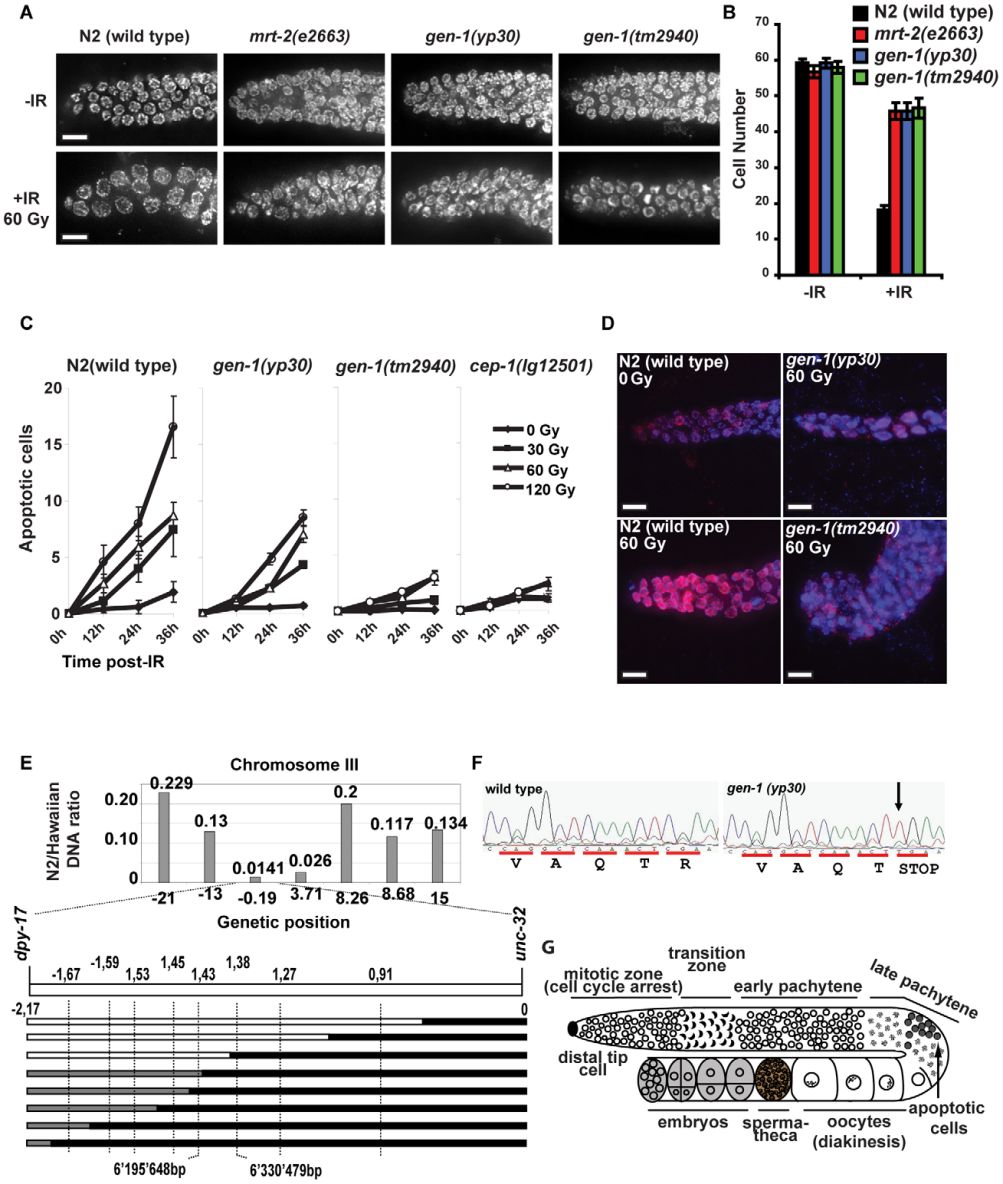
*gen-1* mutants are defective in DNA damage–dependent cell cycle arrest and apoptosis and in positional cloning of GEN-1. (A) Representative pictures of N2-wild type and *gen-1* mitotic germ lines with and without IR treatment (60 Gy). Germ lines were dissected and stained with DAPI. Scale bar 10 µm (B) Statistical analysis of cell cycle arrest. All mitotic germ cells within 50 µm from the distal tip cell were counted (*n* = 7, error bars represent s.e.m.). (C) DNA damage-induced germ cell apoptosis is defective in *gen-1* mutant worms (*n* = 15, error bars represent s.e.m.). Germ cell apoptosis was assayed as described [41]. (D) *gen-1* mutant germ cells fail to arrest in G2. Dissected germlines were stained with the human Cdk-1 phosphotyrosine 15 antibody [42] as a G2 marker (red). (E) Mapping of *gen-1(yp30)*. Linkage to the centre of chromosome III is shown in the upper panel. The ratio of *yp30*/versus “Hawaiian” DNA measured at various single nucleotide polymorphisms. Single recombination events placing *yp30* map position between SNPs located at map position -1,43 and -1,38 are indicated in the lower panel. (F) Chromatograms showing the C to T transition found in the *gen-1 (yp30)* mutant. (G) Diagram of an adult hermaphrodite germ line adapted from [74].

We use the *Caenorhabditis elegans* germ line as a genetic system to study DNA repair and DNA damage response pathways. As part of the *C. elegans* life cycle invariant embryonic cell divisions occur very rapidly. Embryonic cells tolerate a relatively high level of DNA damage using error prone polymerases, possibly a result of natural selection that favours rapid embryonic divisions at the expense of genome integrity [36]. In contrast, the *C. elegans* germ line, which is the only proliferative tissue in adult worms, displays longer cell cycles and is much more sensitive to DNA damaging agents. The gonad contains various germ cell types arranged in a distal to proximal gradient of differentiation (Figure 1G). At the distal end of the gonad cell proliferation occurs in a mitotic stem cell compartment. This compartment is followed by the transition zone where early events of prophase I, such as double strand break generation and the initiation of meiotic chromosome pairing occur. Proximal to the transition zone most germ cells are arrested in the G2 cell cycle phase and reside in meiotic pachytene, where homologous chromosomes are tightly aligned to each other as part of the synaptonemal complex. Germ cells subsequently complete meiosis and concomitantly undergo oogenesis and arrest at the metaphase I stage of meiosis before they are fertilized at the proximal end of the gonad. It takes approximately 20 hours for pachytene stage cells to mature and get fertilized, while the progression of mitotic germ cells till fertilization takes approximately 48 hours [37], [38]. DNA damage such as IR or replication stress, leads to prolonged G2 cell cycle arrest of mitotic germ cells. In addition, late stage meiotic pachytene cells undergo apoptosis in response to DNA damaging agents [39]. DNA damage responses are mediated by components of a conserved DNA damage response pathway (for a review see [40]). Upstream sensors and transducers such as the worm ATR ortholog or components of the 9-1-1 complex promote all DNA damage responses including DNA repair, cell cycle arrest and apoptosis. In contrast, downstream effectors like *cep-1*, which encode the sole primordial p53-like protein of *C. elegans*, are only needed for IR-induced apoptosis [41].

Using unbiased genetic screening and positional cloning approaches we have cloned the *C. elegans* homolog of the human GEN1 HJ resolving enzyme. *C. elegans gen-1* is required for repair of DNA damage-induced DSBs. Surprisingly, *gen-1* mutants are defective in IR-induced cell cycle arrest and apoptosis, indicating that GEN-1 promotes DNA damage signaling. The function of GEN-1 in apoptosis induction is independent of the ATL-1 (*C. elegans* ATR)-dependent induction of the CEP-1/p53 target EGL-1. Our results suggest that GEN-1 is a dual function protein required for the repair of DSBs as well as for DNA damage checkpoint signaling.

## Results

### A screen for *C. elegans* DNA damage response signaling mutants

**Figure 2:**
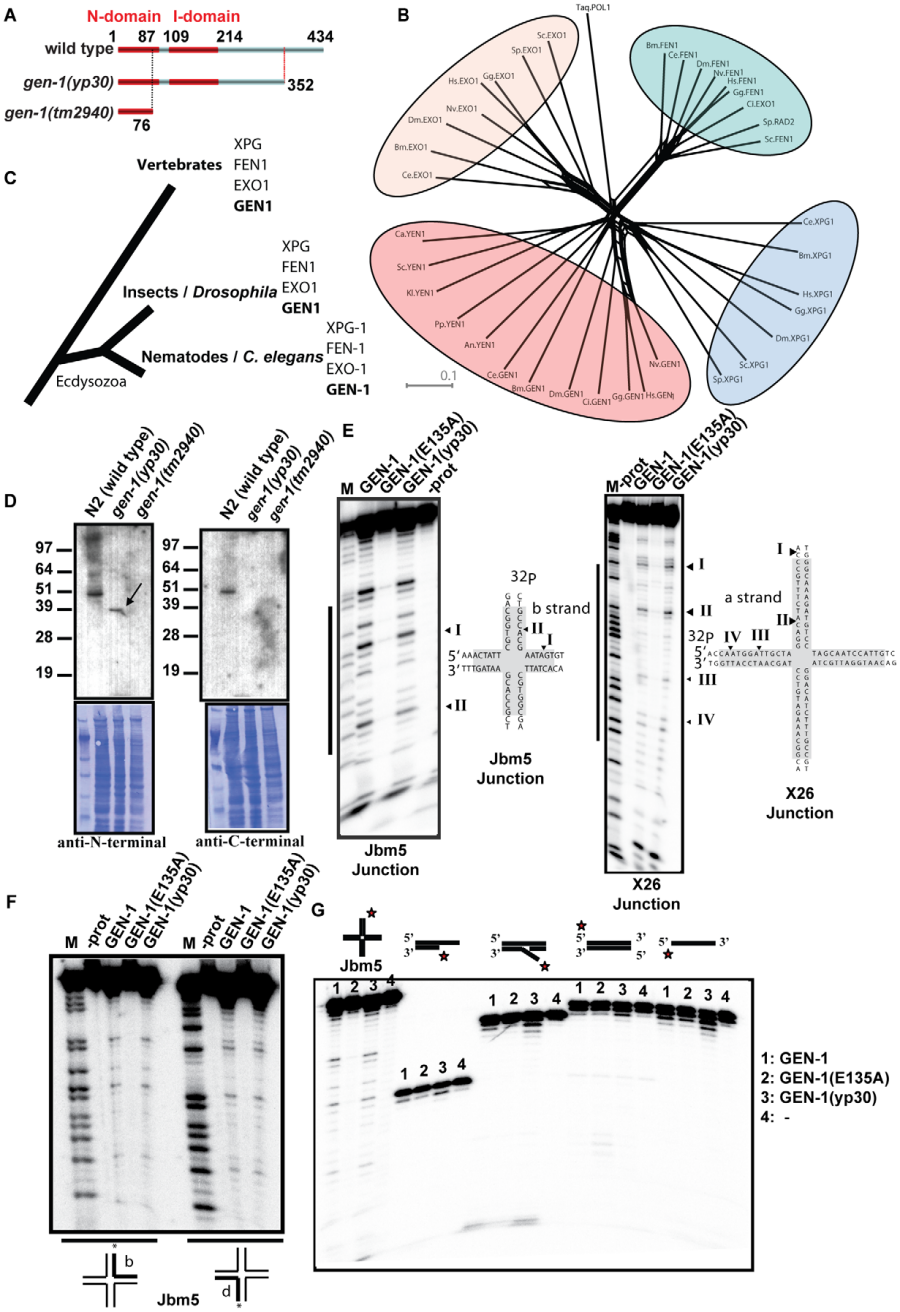
Phylogenetic analysis of GEN-1 and *in vitro* nuclease activity. (A) Diagram showing the GEN-1 domain structure. (B) Unrooted phylogenetic tree of XPG-superfamily members. Bacterial Taq. Pol1 serves as an outgroup. Protein sequences were aligned using Jalview 2.4 employing the MAFT algorithm [83], and the Splitstree program was used to generate the tree shown using the Neighbour Joining Method. The length of the scale bar indicates the lengths of the branches of the phylogenetic trees corresponding to a 10% chance (p 0.1) of replacing an amino acid/site. An, *Aspergillus nidulans*, Bm, *Brugia malayi*, Ca, *Candida albicans*, Ci, *Ciona intestinalis*, Dm, *Drosophila melanogaste*r, Gg, *Gallus gallus*, Hs, Homo sapiens, Nv, *Nematostella vectensis*, Pp, *Pichia pasteuris*, Sc, *Saccharomyces cerevisiae*, Sp, *Schizosaccharomyces pombe*. (C) Four distinct XPG-1 family members occur in all animals examined. The current view of phylogenetic relationships between vertebrates, insects and nematodes is indicated. (D) Affinity purified GEN-1 antibodies (guinea pig) detect a specific band corresponding to the predicted size of GEN-1 and GEN-1 (yp30) (for details Figure S7B, S7C). Equal loading is demonstrated by the Coomassie staining of the membrane. (E) Jbm5 (left panel) and X26 (right panel) junctions, radioactively [5′-^32^P]-labeled on the b and a strand respectively, were incubated at 37°C with the indicated protein (Figure S4A), and the cleavage products analyzed by denaturing gel electrophoresis in polyacrylamide. Lane 1 is a sequence marker for the b stand (left panel). The sequence of the junction is shown, with the homologous section shaded, and cleavage sites are indicated. (F) Symmetry of Holliday junction cleavage by recombinant GEN1. Holliday junction Jbm5, radioactively [5′-^32^P]-labeled on either the b or the d strand was incubated at 37°C with the indicated protein, and the cleavage products were analysed by electrophoresis in polyacrylamide gels under denaturing conditions. M indicates the sequence marker for each respective strand. (G) GEN-1 is specific for Holliday Junctions. Single-stranded, duplex, 3′ overhang and 5′ flap structures were subjected to GEN-1 nucleolytic activity as shown in E. For each reaction, 5 nM of gel purified substrates has been used. For more details see Materials and Methods.

To uncover new genes involved in DNA damage response signaling, we chose an unbiased genetic approach and screened for *C. elegans* mutants hypersensitive to IR and/or defective in DNA damage-induced cell cycle arrest and apoptosis. During *C. elegans* development, the majority of cell divisions occur during embryogenesis. In contrast, germ cell proliferation, which commences with two germ cells at the L1 larval stage, predominates in the following three larval stages and continues in adult worms, where all somatic cells are post-mitotic, but continued germ cell proliferation results in a steady state level of ∼500 germ cells. In order to select for mutants hypersensitive to IR, worms mutagenised with ethyl methane sulphonate (EMS) were irradiated at the L1 stage with 60 Gy of IR. This dose of radiation does not overtly affect germ cell proliferation in wild type worms while mutants hypersensitive to IR display reduced levels of fertility (data not shown). Out of 906 F2 lines screened, 3 mutations (*yp30*, *yp42* and *yp45*) were recovered for the *yp30* complementation group, each of which was derived from an independently mutagenised Po animal. In *C. elegans*, treatment of L4 larvae with IR leads to the activation of a DNA damage response checkpoint pathway that triggers apoptosis of meiotic pachytene stage germ cells, and a transient halt of mitotic germ cell proliferation leading to enlarged cells [39]. This latter phenotype results from continued cellular growth in the absence of cell division. The *yp30* complementation group does not enlarge mitotic germ cells upon IR of L4 larvae, similar to the *mrt-2 (e2663)* checkpoint mutant (Figure 1A and 1B, Figure S1B) [39], and is partially defective in DNA damage-induced apoptosis (Figure 1C). We did not find any further mutants, which were defective in both IR-induced cell cycle arrest and apoptosis like the *yp30* complementation group (data not shown). To show that *yp30* germ cells do indeed fail to arrest cell cycle progression after irradiation, we stained N2 wild type and *yp30* mutants with antibodies against phosphorylated tyrosine-15 CDK-1, which serves as a G2 marker [42], [43]. We found that wild type germ cells arrest in G2, whereas *yp30* germ cells fail to do so (Figure 1D), a finding we confirmed using a YFP::Cyclin B1 fusion construct as a G2 marker (Figure S1A). To clone the gene corresponding to *yp30*, we followed the cell cycle arrest-defective phenotype in backcrossing, SNP-mapping and complementation experiments and positioned *yp30* close to the centre of chromosome III, between *dpy-17* and *unc-32*, to an interval of approximately 135,000 base pairs (Figure 1E, data not shown). Sequencing this interval in *yp30* worms revealed two mutations, one in an intergenic region, and one that leads to a premature stop codon in a gene encoding for a conserved nuclease we refer to as *gen-1* (see below, Figure 1F, Figure 2A). *yp42* and *yp45* also contained the same C to T point mutation as *gen-1(yp30)*, but lacked the intergenic mutation found in *yp30*. Sequencing of the *gen-1* cDNA confirmed the predicted *gen-1* cDNA sequence and the predicted intron-exon structure of *gen-1*, available from Wormbase (http://www.wormbase.org/; data not shown). A *gen-1* deletion allele, *gen-1(tm2940)* (Figure 2A) obtained from the Japanese *C. elegans* knockout consortium, as well as *gen-1* (RNAi), similarly lead to a cell cycle arrest defect upon irradiation (Figure S1B). In addition, the same phenotype was observed in *gen-1(tm2940)*/*gen-1(yp30)* trans-heterozygotes (Figure S1B). Time course and dose response experiments revealed that *gen-1(tm2940)*, *gen-1(yp30)* and *mrt-2(e2663)* worms are equally defective in IR induced cell cycle arrest (Figure S2). Furthermore, *gen-1(tm2940)* is largely defective in DNA damage-induced apoptosis, similar to *cep-1(lg12501)*, a deletion mutant of the *C. elegans* p53-like gene *cep-1* [41], [44] (Figure 1C). In summary, our data reveal that *gen-1* is required for IR-induced apoptosis and cell cycle arrest in *C. elegans* germ cells.

### GEN-1 assignment and phylogenetic relationships

Sequence alignments suggest that GEN-1 is a member of the XPG super-family of nucleases, members of which contain two conserved domains referred to as N and I domains as part of the catalytic centre [45] (Figure 2A, Figure S3). GEN-1 contains putative catalytic residues known to be required for nuclease activity, these are aspartate 77 located in the N domain and glutamate 791 within the I domain of human XPG (Figure S3) [46]. We analyzed all XPG-like genes from fungi, some invertebrates (including other nematodes) and vertebrates, finding that all sequences clustered within four classes of nucleases GEN1, XPG, FEN1 and EXO1, with high probability scores in all species except for fission yeast that does not encode for GEN1 (Figure 2B and 2C). XPG is involved in nucleotide excision repair [47], FEN1 is a flap nuclease involved in lagging strand DNA replication [48], [49], and EXO1 is implicated in genomic stability, telomere integrity [50] as well as DSB end resection [51], [52]. GEN1 was first biochemically characterized based on its flap endonuclease activity in *Drosophila*, and named DmGEN1 (XPG like Endonuclease-1) [53]. A human GEN1 N-terminal fragment was recently purified from HeLa cell extracts, and shown to have robust Holliday junction-resolving activity. Moreover an activity was also found in crude preparations of the budding yeast ortholog Yen1p [33]. The *gen-1(yp30)* mutation leads to the expression of a C-terminally truncated protein that does not affect the putative catalytic centre (Figure 2A and 2D). In contrast, the *tm2940* deletion is predicted to eliminate the majority of the I domain and is likely to be a null allele, as anti-GEN-1 antibodies detected GEN-1 protein for wild-type and *gen-1(yp30)* strains but not for *gen-1(tm2940)* (Figure 2A and 2D, Figure S7B and S7C).

To determine if *C. elegans* GEN-1 exhibits Holliday junction-resolving activity *in vitro*, as predicted from homology to the human GEN1, recombinant wild type GEN-1, GEN-1 (yp30) and an E135A mutant were expressed and purified, the latter bearing a mutation in one of the putative nuclease active site residues (Figure S4A). A Holliday junction-resolving enzyme should symmetrically cleave Holliday junctions and be specific for four-way DNA junctions. We tested for GEN-1 nuclease activity on two four-way DNA junctions. Jbm5 contains a 12 base pair homologous core through which the branch point can migrate [54], and X26 contains a 26 base pair core and bears sequences unrelated to Jbm5 [33]. Using both four-way junction substrates we observed specific cleavage using GEN-1 and GEN-1 (yp30) recombinant enzymes (Figure 2E). Using both substrates the same cleavage pattern was observed on opposite strands as expected from symmetry (Jbm5, Figure 2F, data not shown). To confirm structural specificity towards four-way DNA junctions, we tested whether *C. elegans* GEN-1 showed specific nuclease activity towards a variety of other substrates, including single-stranded, blunt double-stranded DNA, a dsDNA substrate with a 3′ single-stranded overhang, and a 5′ flap structure. We observed no specific cleavage of any of these substrates with *C. elegans* GEN-1 (Figure 2G, Figure S4C). Comparing the cleavage to that generated by the human GEN1 (comprising amino acids 1-527), we find that the major Jbm5 cleavage product resulting from incubation with human GEN1 also occurs upon incubation with the *C. elegans* protein (Figure S4B). Human GEN1 also showed an activity towards 5′ flap structures as reported previously [33] (Figure S4C). The enzymatic activity of the recombinant *C. elegans* enzyme is relatively low; we thus cannot exclude the possibility that *C. elegans* GEN-1 also shows a 5′ flap activity, albeit we did not observe such an activity in overexposed gels and multiple repeat experiments. We speculate that the low activity of recombinant *C. elegans* GEN-1 might be due to improper folding. Alternatively, the worm nuclease might require post-translational modifications, interacting proteins or activation by proteolytic cleavage to become fully activated as a HJ resolving enzyme, thereby preventing us from undertaking a more thorough analysis of its biochemical properties at the present time. Nevertheless, the cleavage introduced into the four-way junction by *C. elegans* GEN-1 as well as the orthologous relationship to human GEN1 and budding yeast Yen1p, is consistent with GEN-1 being a junction-resolving enzyme in *C. elegans*.

### GEN-1 is required for DNA double-strand break repair

**Figure 3:**
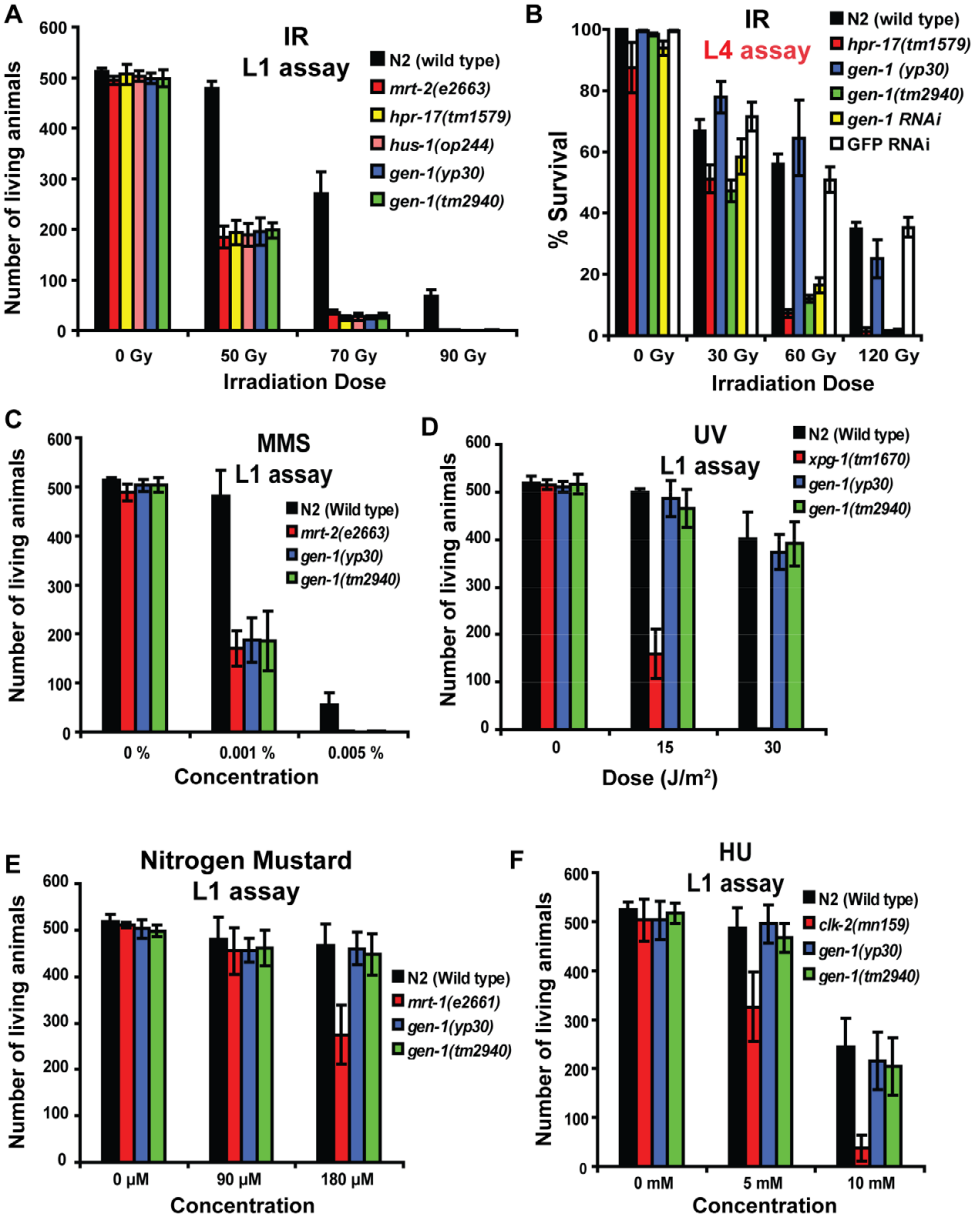
Sensitivity of *gen-1 (tm2940)* and *gen-1 (yp30)* in response to DNA damaging agents. (A) L1 stage worms sensitivity assay to IR, (B) L4 stage worms sensitivity assay to IR. (C) MMS, (D) UV irradiation, (E) nitrogen mustard, and (F) HU exposure of L1 stage worms sensitivity assay. Assays were performed as described in Materials and Methods. Error bars represent s.e.m.

A Holliday junction-resolving activity is likely to be required for meiotic recombination, and a defect in this activity is predicted to result in embryonic lethality due to random autosome segregation in meiosis [55]. We can exclude such a defect as *gen-1(tm2940)* worms propagate as wild type, and fail to exhibit embryonic lethality in the absence of genotoxic stress (Figure 3A (0 Gy)). Furthermore, we did not observe an enhanced incidence of XO males, a phenotype that would indicate defects in meiotic chromosome pairing or recombination of the X chromosome (Table 1) [56].

**Table 1:** Incidence of males and brood size in N2 wild type and *gen-1* mutants.

Genotype	Brood Size	% Males
N2 (*n* = 10)	272±18	0.15±0.08
*gen-1(yp30)* (*n* = 10)	271±22	0.12±0.04
*gen-1(tm2940)* (*n* = 10)	296±15	0.14±0.04

Most *C. elegans* mutations of DNA damage checkpoint genes, such as *hpr-17*, encoding for the Replication Factor C homolog of *S. pombe* Rad17, are also considered to be required for DNA DSB repair, as the corresponding mutants are hypersensitive to IR [39]. Two assays allow for testing the IR sensitivity of cells residing in different germ line compartments. In the “L1” IR survival assay that corresponds to the screening conditions we initially used to isolate *yp30* as an IR sensitive mutant, the sensitivity of mitotic germ cells is evaluated by irradiating L1 larvae and by assaying for sterility of the resulting adults. The extent of sterility is scored by counting the number of worms in the following generation. Upon irradiation of L1 larvae, *gen-1(yp30)* and *gen-1(tm2940)* mutants were equally hypersensitive to IR, similar to *hus-1(op244)*, *mrt-2(e2663)* and *hpr-17(tm1579)* positive control strains (Figure 3A). To assess whether GEN-1 might also be required to repair DNA damage induced by methyl methane sulonate (MMS) treatment, we tested for MMS sensitivity in a manner analogous to the assay for radiation. MMS leads to double-strand breaks when DNA replication forks encounter alkylated bases and mutants defective in recombinational repair are MMS sensitive [57]. We found that *gen-1(tm2940)* and *gen-1(yp30)* were MMS hypersensitive (Figure 3C). In contrast to various control mutants with DNA repair defects, *gen-1* mutants were not hypersensitive to UV irradiation, which causes lesions predominately repaired by excision repair (Figure 3D). Neither DNA cross-linking by nitrogen mustard, which is largely repaired by the DNA interstrand cross link pathway, nor hydroxyurea which slows DNA polymerase processivity by nucleotide depletion, led to hypersensitivity in *gen-1* mutants (Figure 3E and 3F). To corroborate our results, we also employed the L4 irradiation assay [58]. In the “L4” IR assay, the sensitivity of meiotic pachytene cells is determined by measuring survival of embryos that are produced ∼20 hours after irradiation; these embryos are derived from pachytene cells that are arrested in the G2 cell cycle stage for more than 10 hours prior to completing meiosis and oogenesis. We found that both *gen-1(tm2940)* and *gen-1*(RNAi) are as IR-sensitive as the *hpr-17(tm1579)* deletion, whereas *gen-1(yp30)* pachytene germ cells were not sensitive to IR (Figure 3B). A similar response profile was found in response to MMS treatment (Figure S5A), while no enhanced sensitivity was found in response to UV, nitrogen mustard, or hydroxyurea (Figure S5B and S5D). Thus, the *gen-1(yp30)* allele, which results in a C-terminally truncated protein that retains nuclease activity *in vitro*, elicits IR- and MMS-induced hypersensitivity for mitotic germ cells of L1 larvae, whereas a null *gen-1* mutation displays additional hypersensitivity to these agents in L4 germ cells arrested in pachytene. Our results suggest that the signaling function of GEN-1 is likely conferred by the C-terminus of GEN-1, given that the *gen-1 (yp30)* C-terminal truncation mutants as well as the *gen-1 (tm2940)* deletion are defective in checkpoint signaling, whereas *gen-1(yp30)*, which retains nuclease activity that may directly promote DNA repair in mitotic germ cells. The differential sensitivity of the *gen-1 (yp30)* allele in L1 and L4 survival assays likely reflects the fact that checkpoint-induced cell cycle arrest contributes to the survival of mitotic germ cells to IR. Furthermore, *gen-1 (yp30)* is only partially defective for IR-induced germ cell apoptosis (Figure 1C).

**Figure 4:**
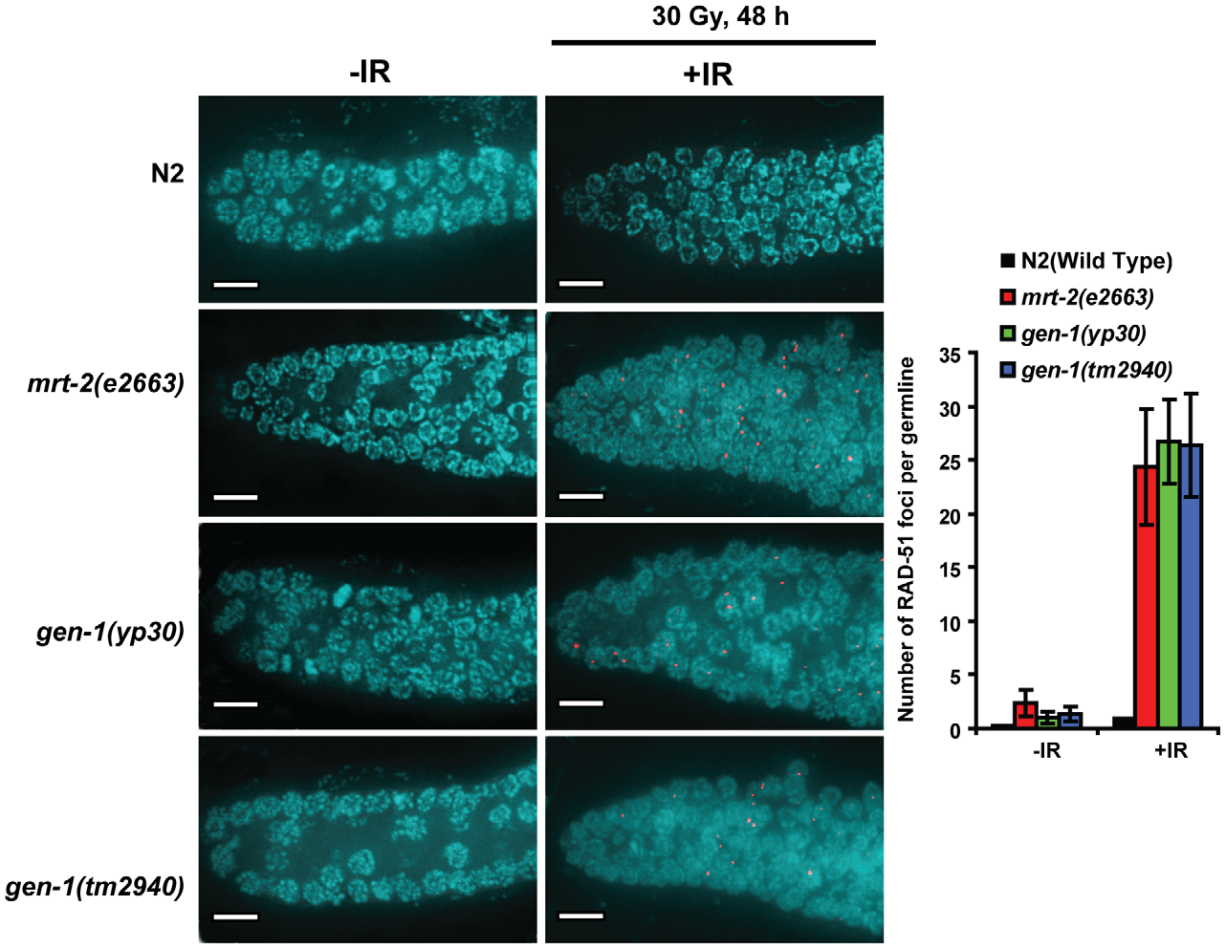
Persistent RAD-51 foci in *gen-1(tm2940)* and *gen-1(yp30)* worms. Rad-51 foci were scored 48 h post IR with 30 Gy as described [82]. A layer of only five z-stacks is displayed for clarity. DAPI and RAD-51 correspond to blue and red staining respectively. A quantification of foci per mitotic germ line is shown in the right panel. *n* = 5, error bars represent s.e.m. Foci were counted by projecting all the z-stacks using SoftWorks. Scale bar is 10 µm.

**Figure 5:**
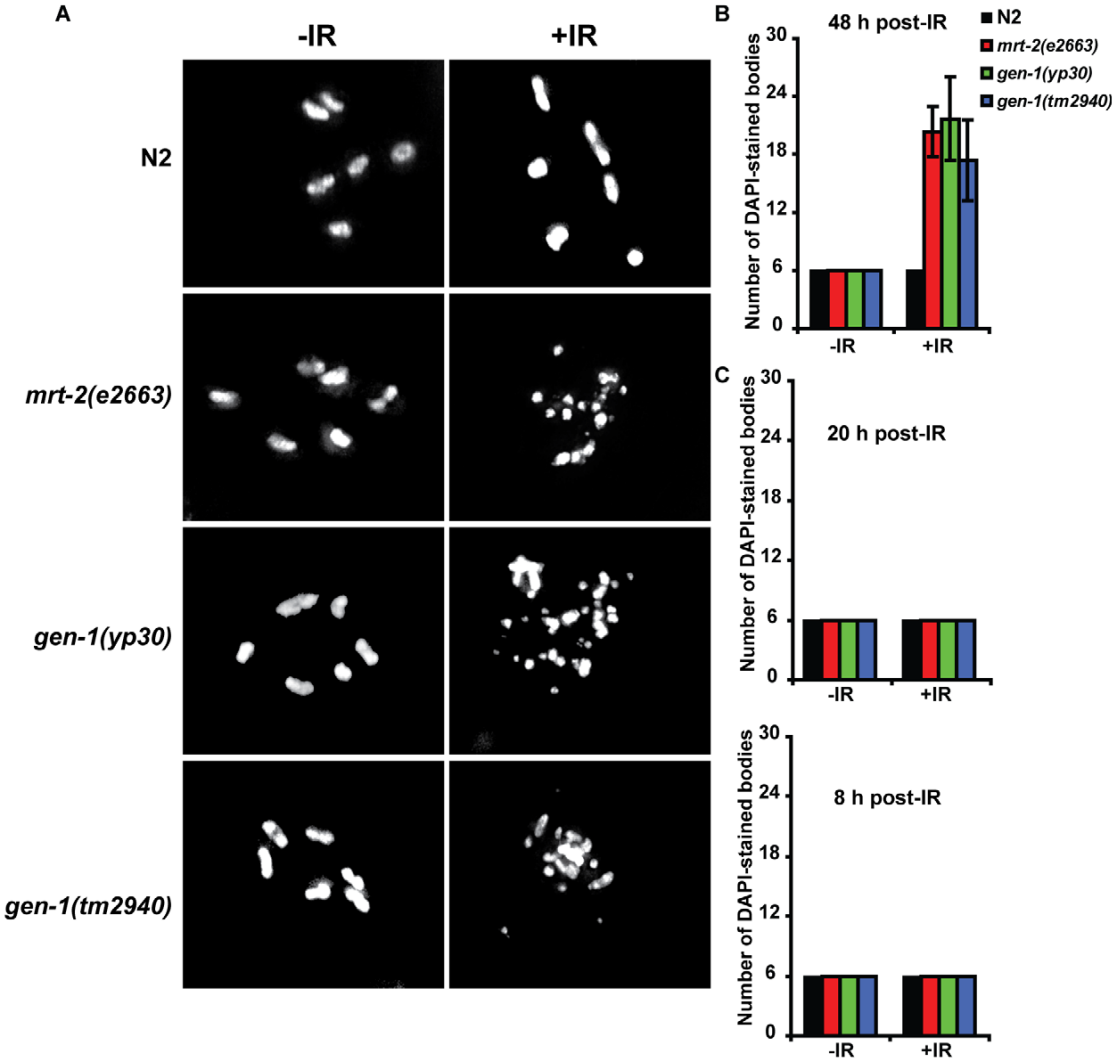
Chromosome fragmentation assay. (A) L4 worms were irradiated with 90 Gy of IR and dissected 48 h later. Scale bar is 5 µm. (B) A quantification of the extent of chromosome fragmentation (*n* = 12). (C) No chromosome fragmentation is observed 20 h (top panel) and 8 h after irradiation (lower panel).

To test if the IR sensitivity phenotypes of *gen-1* mutants correlate with persistence of DSBs, we assayed for RAD-51 foci. At doses where multiple DSBs per cell are generated, the number of persistent RAD-51 foci in mitotic germ cells of *mrt-2(e2663)* and both *gen-1* mutants is higher as compared to wild type, indicating a DSB repair defect (Figure 4). To directly confirm whether IR leads to increased DNA double-strand breakage in *gen-1* mutant worms we directly assayed for chromosome fragmentation after irradiation with 90 Gy. As shown previously [59], 48 hours after irradiation of mitotic germ cells (at the L4 stage) the diakinesis chromosomes of resulting *mrt-2(e2663)* oocytes were fragmented. In contrast, IR-induced damage was repaired in wild type, where oocyte chromosomes appear as 6 morphologically intact condensed DAPI stained structures (Figure 5A and 5B). Chromosome fragmentation for both *gen-1* mutants was as strong as that observed for the *mrt-2* positive control, indicating a defect in DSB repair. This chromosome fragmentation phenotype was not observed as a consequence of irradiating pachytene stage cells and observing corresponding oocytes ∼8 hours and ∼20 hours after IR (Figure 5C). Given that *gen-1(tm2940)* and *gen-1(yp30)* are equally defective in repairing diakinesis chromosomes 48 hours after irradiation we consider it likely that the checkpoint functions of *gen-1* (and *mrt-2*) in mitotic germ cells contribute to DSB repair. Given that DSBs inflicted in pachytene cells are repaired in *gen-1* and *mrt-2* mutants while this is not the case for DSBs in mitotic germ cells there might be a stronger requirement of GEN-1 and MRT-2 for DSB repair in mitotic germ cells.

### GEN-1 may act in parallel to ATM and the 9-1-1 complex to facilitate DSB repair

**Figure 6:**
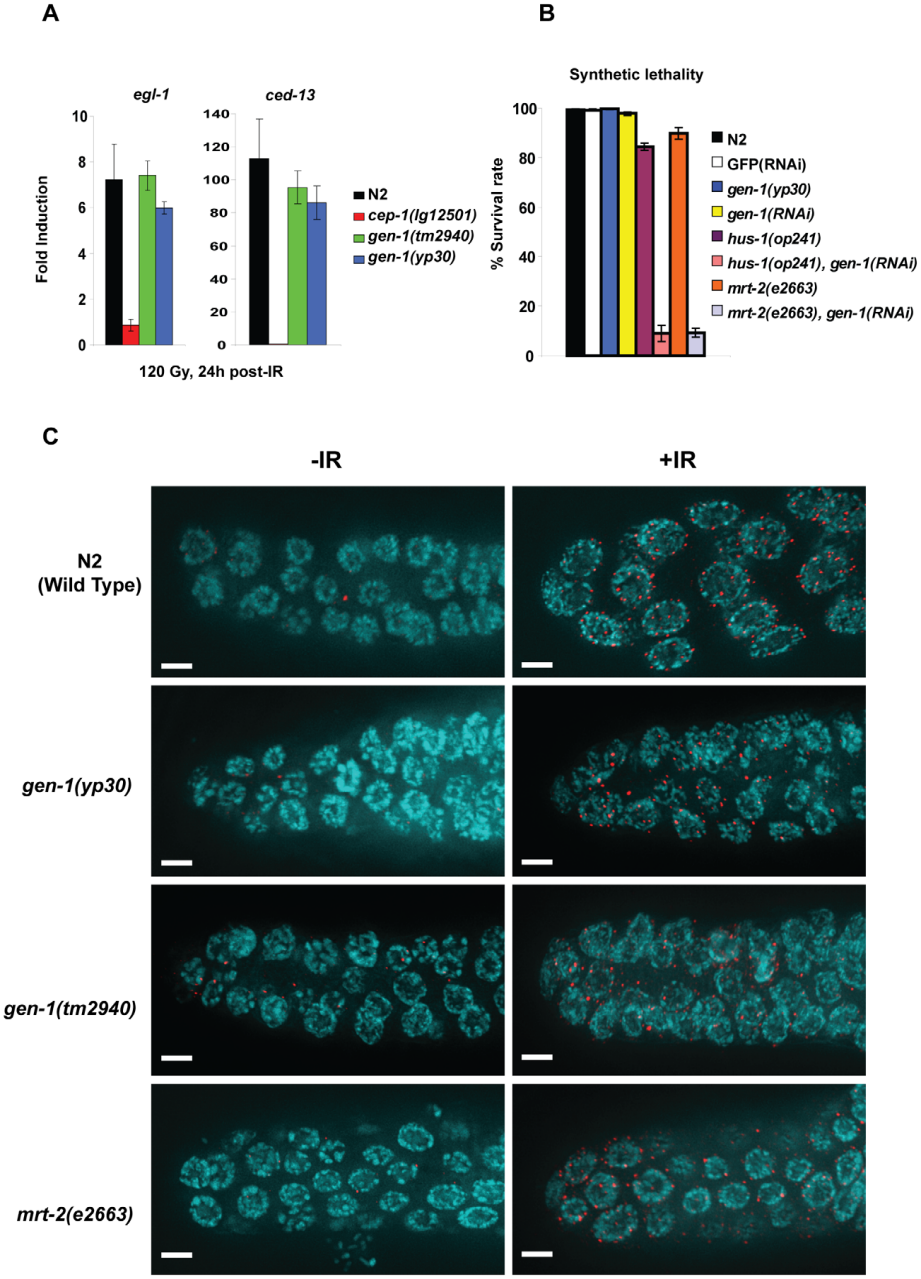
*gen-1* acts in a non-canonical DNA damage checkpoint pathway. (A) *cep-1* mediated transcriptional induction of *egl-1* and *ced-13*. Quantitative RT-PCRs were performed as described [63], induction compared to unirradiated N2 wild type is shown (B), *gen-1* RNAi leads to synthetic lethality in conjunction with *hus-1(op241)* and *mrt-2(e2663)* mutations. Percent (%) survival indicates the number of eggs hatched and grown to adulthood. RNAi against GFP was used as negative RNAi feeding control. (C) Phospho-Chk-1 antibody staining 6 h after IR (60 Gy) treatment of the indicated genotypes. Scale bar is 10 µm.

We next wished to determine if *gen-1* acts in a known pathway promoting the repair of DSBs. We first examined if *gen-1* affects non-homologous DNA end joining. In *C. elegans* DNA end joining is predominantly used in somatic cells. Worms defective in DNA end joining genes such as *lig-4*, *cku-70* and *cku-80* show a reduced pace of development upon IR of embryos [59]. We found that neither of the *gen-1* mutants exhibited any such somatic developmental delay (Figure S7A). The strong IR-sensitivity and the defect in checkpoint-dependent cell cycle arrest and apoptosis of *gen-1(tm2940)* is reminiscent of the phenotype of mutations in upstream DNA damage signaling factors such as the *C. elegans* 9-1-1/Replication Factor C-like complex members *hus-1* and *mrt-2 (S. pombe rad1)*. Given that mutations of genes encoding for the 9-1-1 complex lead to telomere replication defects [60], we asked if sterility in later generation worms caused by progressive telomere attrition occurs in *gen-1(tm2940)*. We failed to observe such an effect, further indicating that *gen-1* is not part of the *mrt-2* epistasis group (Figure S8A). To investigate further how GEN-1 affects DNA damage responses, we depleted *gen-1* in *hus-1* or *mrt-2* mutant backgrounds. RNAi depletion of *gen-1* in *hus-1* or *mrt-2* mutant strains leads to synthetic lethality (Figure 6B). We confirmed this synthetic lethality by *gen-1 hpr-17* double mutant analysis (Figure S8B). *hpr-17* encodes for the 9-1-1 clamp loader and is part of the *mrt-2* epistasis group. As expected, *gen-1* RNAi in a *mrt-2(e2663)* background led to an increased number of RAD-51 foci as compared to *gen-1* RNAi in wild type worms and to the *mrt-2(e2663)* mutant in mitotic germ cells (Figure S9). In contrast, *gen-1(yp30)*, which is checkpoint-defective but encodes a protein that can promote HJ resolution *in vitro*, did not cause synthetic lethality when combined with an *hpr-17* mutation, nor did it exacerbate the radiation hypersensitivity phenotype of *hpr-17* (Figure S8C). These results therefore suggest that the DNA repair function of GEN-1 may act redundantly with the 9-1-1 complex to repair DSBs occurring during normal DNA replication.

**Figure 7:**
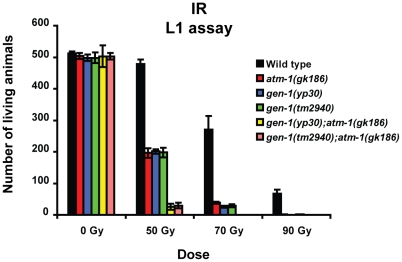
L1 stage worms IR sensitivity assay with *gen-1* and *gen-1 atm-1* double mutants. Assays were performed as described in Figure 3. Error bars represent s.e.m.

We next wished to determine genetic interactions between GEN-1, ATR and ATM PI3-like kinases, which are predicted to act upstream of the 9-1-1 complex in DNA damage signaling. Given that an *atl-1(tm853)* deletion leads to excessive genome instability in germ cells and concomitant sterility [61], we could not assess the possibility of enhanced IR sensitivity in *gen-1 atl-1* double mutants. In contrast, *C. elegans atm-1* plays a minor role in DNA damage signaling and *atm-1(gk186)* results in partial defect in IR-induced cell cycle arrest and apoptosis [62]. Consistent with this notion, we found that the *atm-1(gk186)* deletion is not hypersensitive to IR when subjected to the L4 IR survival assay, and that the IR sensitivity is not enhanced by the *gen-1(yp30)* mutant (Figure S10). In contrast, the *atm-1(gk186)* mutant is sensitive to IR in the L1 assay, and IR sensitivity is enhanced in combination with both *gen-1(tm2940)* and *gen-1(yp30)* (Figure 7).

In summary, our results suggest that *gen-1* might act in parallel to *atm-1* for repairing mitotic germ cells affected by DNA double-stranded breaks.

### GEN-1 may act in parallel to the chk-1 cep-1/p53 checkpoint pathway

Given that *gen-1* encodes for a nuclease, we wanted to eliminate the possibility that GEN-1 might also be required for the processing of DSBs to generate single-stranded DNA overhangs, which would be coated by RPA1 and lead to the ATRIP-dependent activation of ATR in mammalian cells [2]. We thus tested whether IR-dependent RPA-1 loading is compromised in *gen-1(tm2940)* worms. We found that the sequential accumulation of RPA-1 (green) and RAD-51 (red) foci does not significantly differ between wild type and *gen-1(tm2940)* worms, indicating that the initial steps of DSB processing occur normally in *gen-1* mutants (Figure S6A). These results are corroborated by our finding that GEN-1 does not cleave double-stranded substrates or substrates with 3′ single stranded overhangs *in vitro* (Figure 2G).

To monitor the activation of the *C. elegans* ATL-1/ATR-mediated DNA damage checkpoint pathway in *gen-1(tm2940)* mutants, we analyzed the IR-induced transcriptional induction of the pro-apoptotic BH3-only domain encoding genes *ced-13* and *egl-1*. The induction depends on the *C. elegans* CEP-1 p53-like transcription factor [63], and on upstream DNA damage response genes including *atl-1 (C. elegans* ATR), c*lk-2*, *hus-1* and *mrt-2* [64]. *egl-1* and *ced-13* were induced to near-normal levels for *tm2940* and *yp30* alleles of *gen-1*, while no induction occurred in a *cep-1(lg12501)* background (Figure 6A). Thus, the apoptotic signaling function of GEN-1 acts in parallel to the canonical *C. elegans* DNA damage response pathway necessary for *egl-1* induction. To further support this notion, we cytologically probed for the activation of CHK-1, which is required for IR-induced cell cycle arrest and apoptosis in *C. elegans* [65]. To this end we employed an antibody against a conserved CHK-1 phosphopeptide that includes serine 345 [36], [66]. Phosphorylation of this residue in response to DNA damage depends on ATR and ATM kinases and leads to Chk1 activation in mammals [67]–[69] and occurs in response to ATL-1/ATR activation in *C. elegans* [36], [66]. CHK-1 phosphorylation is increased in response to IR in cell cycle arrested cells (Figure 6C, top panel), both in wild type as well as in *gen-1* mutants, further substantiating the notion that the checkpoint signaling function of GEN-1 might act in parallel to the canonical pathway. Interestingly, CHK-1 phosphorylation also occurs in the *mrt-2 (e2663)* mutant (Figure 6C). This data indicates that ATM/ATR is not fully dependent on *mrt-2*, consistent with the reduction as opposed to the complete alleviation of CEP-1 dependent transcription in this mutant [64]. Thus our results suggest that *gen-1* and *mrt-2* act in parallel pathways needed for checkpoint signaling similar to their roles in DSB repair.

## Discussion

**Figure 8:**
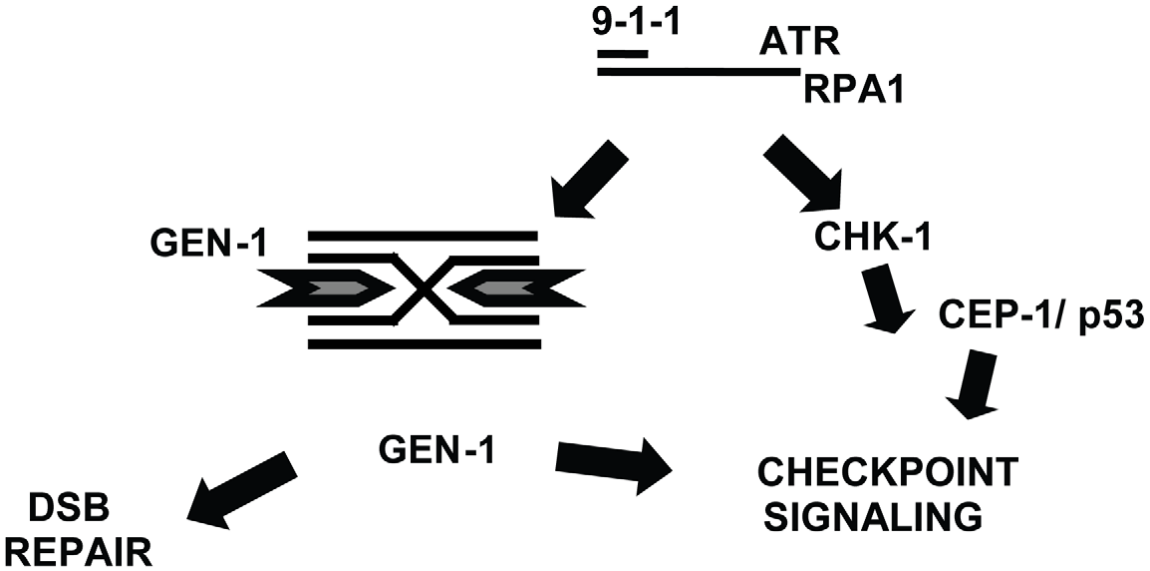
Model. A processed DSB with a single stranded tail coated by RPA1 and ATR is indicated at the top of the panel. Signaling via the 9-1-1 complex, Chk-1 and CEP-1/p53 is indicated in the right. A Holliday junction is depicted on the left and the predicted symmetrical cleavage activity of GEN-1 is indicated by two arrows.

We have discovered that the deficiency of GEN-1 results in DNA damage signaling defects (Figure 8). Neither cell cycle arrest of mitotic germ cells, nor apoptosis induction of meiotic pachytene cells occurs in response to DNA damage in *gen-1* mutants. These defects are as severe as those observed in known *C. elegans* checkpoint mutants such *atl-1*, the worm ATR homolog [61], *clk-2* [70] and mutants affecting components of the *C. elegans* 9-1-1 complex [60], [71], [72]. Intriguingly, we find that the apoptosis defect conferred by a mutation in *gen-1* does not result from the ATR-, CLK-2- and 9-1-1 complex-dependent activation of the primordial worm p53-like protein CEP-1 (Figure 8) [41], [44]. The two known CEP-1 target genes *egl-1* and *ced-13*, whose transcriptional activation confers the inhibition of the anti-apoptotic Bcl2 like protein CED-9, are normally induced [63]. Thus, the signaling function of GEN-1, which promotes apoptosis in meiotic germ cells, appears to be in a pathway acting in parallel or downstream of the canonical DNA damage response pathway that activates CEP-1/p53. Analogous results have been observed for *C. elegans sir-2.1* histone deacetylase, as well as for *hyl-1* and *lagr-1* ceramide synthase mutants, where CEP-1 targets are upregulated in response to DNA damage even though germ cell apoptosis fails to occur [73], [74]. Thus, *gen-1*, *sir-2.1*, *hyl-1* and *lagr-1* may define components of a DNA damage response pathway that functions in parallel to the pathway, which needs CHK-1 and the 9-1-1 complex. How these pathways are integrated remains to be elucidated. We speculate that GEN-1 may facilitate DSB repair by coordinating cell cycle progression with HJ resolution.

Our evidence that *C. elegans* GEN-1 acts as a HJ-resolving enzyme is supported by the biochemical characterization of its human and yeast orthologs [33]. Given the orthologous relationship with *C. elegans* GEN-1 and our biochemical evidence, it is likely that *C. elegans* GEN-1 can act as a HJ-resolving enzyme *in vivo*. The active form of GEN-1 purified form HeLa cell extracts is a C-terminal truncation [33]. We analyzed multiple preparations of full length and truncated versions of *C. elegans* GEN-1 but could only obtain weak nuclease activity on mobile HJ substrates. Nevertheless, this activity is lost if one of the putative active site residues was mutated, and it was specific for Holliday junction substrates. Thus, the nuclease function of *C. elegans* GEN-1 during DSB repair may involve HJ resolution. Future studies may refine our understanding of the substrate specificity of GEN-1.

Our results point towards the possibility that the completion of HJ resolution in response to DNA damage-induced DSBs might be monitored by GEN-1, which might act both as a Holliday junction-resolving enzyme as well as a DNA damage signaling molecule (Figure 8). A dual function enzyme that catalyses a late step of recombination and plays a role in checkpoint signaling could provide a mechanism to suppress cell cycle progression to allow for the repair of the majority of DNA double-strand breaks before cell cycle progression resumes. The signaling function of GEN-1 is likely conferred by the C-terminus of GEN-1. The C-terminus of GEN-1 may therefore interact with known or novel DNA damage signaling molecules that function to promote DSB repair in mitotic germ cells.

At the moment we can only speculate about the nature of the RAD-51 foci that persist in *gen-1* mutants. Some of these RAD-51 foci might correspond to recombination intermediates resulting from failure of specific types of checkpoint-mediated DNA repair. Alternatively, these foci might be the consequence of initial unrepaired DNA damage that result in double-strand breakage once unrepaired DNA is replicated when cells resume cell division.

Given the specificity with which GEN-1 processes HJ structures *in vitro*, it is surprising that GEN-1 does not have any obvious function in meiotic recombination. One candidate for a *C. elegans* meiotic HJ-resolving enzyme might be the Him-18/SLX4/Mus312 SLX1 nuclease complex. The rate of meiotic recombination is significantly reduced in *Drosophila mus312* mutants [75], and the human SLX1/SLX4 complex has recently been shown to have HJ resolution activity *in vitro* [28]–[31]. Further, lack of a role for GEN-1 in meiotic crossover resolution is consistent with recent evidence that *Drosophila* and *C. elegans him-18/slx-4* may promote meiotic Holliday junction resolution [31], [32]. Additional proteins implicated in resolving meiotic HJ initially in fission yeast and fruit flies are Mus81 and Xpf1, respectively [21], [75]. Further, the combined activities of Bloom's helicase and topoisomerase III have been shown to dissolve HJ independently of canonical junction-resolving activities *in vitro* [13], [14]. However, the meiotic defects of the C. *elegans mus-81*, *xpf-1 or him-6* Bloom's orthologs are not overtly enhanced by the *gen-1 (tm2940)* mutation (Simon Boulton, personal communication). Collectively, the absence of a meiotic defect of *gen-1* together with the lack of strong synthetic effects with candidate meiotic HJ resolving enzymes, strongly suggests that *C. elegans* GEN-1 does not play a central role in this process.

Although different species vary in their precise DNA double-strand break response strategies, and various cell types are likely to utilize different DSB repair pathways preferentially, basic regulatory complexes and processes tend to be conserved. *C. elegans* GEN-1 plays an essential role in responding to DSBs, but it is inert in budding yeast [34] and has apparently been lost during evolution of fission yeasts. In addition to DNA end-joining, which does not require HJ resolution, DSBs can be repaired without a HJ resolution step by DNA synthesis-dependent strand annealing [57], [76], [77]. We speculate that this may be related to an inherent redundancy in DNA double-strand break repair pathways in diverse organisms, and perhaps within various tissues of the same organism. Indeed, our staining for RAD-51 foci indicates that most DSBs are repaired in *gen-1* mutants, likely by a combination of the above mentioned recombinational repair pathways and non-homologous end joining, but that a fraction of these breaks persists 48 hours after IR (Figure 4). Such a scenario is in line with recent data suggesting that a subset of persistent DBSs is repaired by distinct DSB repair pathways [59], [78]. In mammals these persistent foci are associated with heterochromatin and their repair specifically requires ATM [79], which may be consistent with the enhanced DNA damage response defects observed for *atm-1;gen-1* double mutants. Thus, GEN-1 might be involved in DSB repair processes that are redundant and therefore hidden within DSB response networks in some organisms.

It has recently been reported that GEN1 is absent in ovarian and colon cancer cell lines, suggesting that GEN1 is required for maintaining genome stability in human cells [80]. Thus, GEN1 might join the number of genes involved in recombinational repair such as BRCA1, BRCA2, and FANCJ/BACH, mutation of which is associated with cancer. Deletion of these genes does not result in cellular lethality, but affected cancer cells are uniquely sensitive towards DNA damaging agents allowing their selective eradication. Redundant mechanisms involved in resolving HJ structures might be particularly amenable to such synthetic lethal approaches. Our finding that *gen-1* is synthetically lethal with mutations in known DNA damage sensors and repair proteins encoded by the 9-1-1 complex suggests one such mechanism.

Overall, our results show that GEN-1, a protein previously implicated in HJ resolution, possesses dual function that potentially couples DNA repair and DNA damage signaling.

## Materials and Methods

### *C. elegans* strains and maintenance

Worms were maintained at 20°C on NGM agar plates seeded with *E. coli* strain OP50 as previously described [81], unless otherwise indicated. Alleles are all described in the CGC *C. elegans* stock center. We generated the following strains as part of this study TG1043 *gen-1(yp30)III*; TG1540 *gen-1(tm2940)III*; TG765 *cep-1(lg12501)II*; TG1236 *gen-1(yp30) unc-32(e189)III*; TG1237 *gen-1(yp30) dpy-17(e164)III*; TG1233 *hpr-17(tm1579)II*; TG771 *hus-1(op244)I*; TG545 *hus-1(op241)I*; TG1503 *hpr-17(tm1579)II: gen-1(tm2940)III*; TG1502 *gen-1(yp30)III*, *opIs76(CYB-1::YFP)*; TG1064 *gen-1(yp42)III*; TG1060 *gen-1(yp45)III; TG1565 xpg-1(tm1670)I*; RB964 *cku-80(ok861)III*; TG190 *clk-2(mn159)III*; VC381 *atm-1(gk186)I*.

DNA damage-induced apoptosis and L4 radiation hypersensitivity (rad) assays were performed as described [39]. For γ-irradiation a Cs137 source (2.9 Gy/min, IBL 437C, CIS Bio International) was used.

### Protein expression and GEN-1 antibody production

6x-histidine tagged full length GEN-1 (pGA343) was expressed in BL21(DE3) CodonPlus cells, recovered from inclusion bodies using BugBuster (Novagen), solubilised in Urea buffer and purified with Ni-NTA following manufacturer's instructions (Qiagen). One guinea pig was immunized (BioGenes GmbH) and antibodies were affinity-purified from the final bleeding using Maltose Binding Protein (MBP) tagged protein. N- and C-terminus GEN-1 (fragments 1-136 and 356-434 respectively) tagged with MBP (pGA346 and pGA348) were purified using an amylose resin column (New England BioLabs). For affinity purification, proteins were covalently linked to AffiGel 15 (Bio-Rad).

### Immunostainings

Immunostaining experiments were performed as described [74]. Primary antibodies used were rabbit anti-RAD-51 (1/200 dilution) as described [82], rat anti-RPA-1 (1/100 dilution), rabbit anti-Cdk1 (pTyr15) Calbiochem, 219440 (1/50 dilution) and rabbit anti-P-CHK1 (P-CHK1 Ser 345: sc-17922, Santa Cruz, 1/50 dilution). Secondary antibodies used were Cy3 labeled anti-rabbit (Jackson Immunochemicals) 1/1000 dilution and FITC anti-rat (Jackson Immunochemicals) 1/200 dilution.

### RPA-1 antibodies

*C. elegans* full-length ORF of *rpa-1* was cloned into pMAL-2c vector (New England Biolabs) and expressed in BL21 (DE3) cells. The MBP-tagged protein was purified on an amylose resin following the manufacturer's instructions (New England Biolabs) and used to immunize one rat (Eurogentec animal SAOI.1). The same purified protein was covalently linked to AffiGel-15 (BioRad) and used to affinity-purify antibodies from the final bleed.

### L1 genotoxic assays

L1 larvae stage worms were sorted from a growing population using an 11 µm filter (Millipore NY11) and treated with the indicated genotoxic agents. To test MMS and Nitrogen mustard sensitivity, worms were incubated with the indicated concentration of mutagen for 12 hours in M9 buffer. UV irradiation was performed by the XL-1000 Spectrolinker UV-C light source. 5 L1 stage worms in the P0 generation were plated onto a single plate. The number of living worms (post the L1 stage) present in the F1 generation within 48 hours of (untreated) P0 worms reaching the L4 stage was counted using a dissection microscope. For hydroxyurea (HU), L1 worms were plated on 1x NGM plates supplemented with the indicated compounds and the living adult worms corresponding to the F1 generation were established similarly. Experiments were done at least in triplicate.

### RNAi feeding

RNAi feeding was done as described with exception of using 1 mM IPTG [58].

### Cleavage assays

Recombinant proteins (50 nM) were added to 2 nM (Figure S4) or 5 nM (Figure 2E–2G) of the indicated substrates all of which were 5′ [^32^P]-labelled on one strand in 10 mM Tris-HCl pH 8.0, 10 mM NaCl, 10 mM MgCl_2_, 0.1 mg/ml BSA, 0.1 mg/ml calf thymus DNA, 1 mM DTT and 1 M NDSB 201. Samples were incubated at 37°C for 30 min or overnight, and the reaction terminated by addition of EDTA. Cleavage products were analysed by electrophoresis in 12% polyacrylamide gels containing 8 M urea. Gels were dried and imaged using a Fuji BAS 1500 phosphorimager.

### Substrates

For the nuclease assays, substrates were generated as described [33], the following oligonucleotides were used, sequences are shown 5′ to 3′:

Single-stranded:

-1971: CGCTCTAGAGCGGCTTAGGCTTAGGCTTAGGCTTA

Double-stranded (annealing 1971 and 1972):

-1972: TAAGCCTAAGCCTAAGCCTAAGCCGCTCTAGAGCG

5′ Flap (annealing A-Flap, B and C)

3′ Flap (annealing B-Flap, A and C)

-A-Flap: ATGTGGAAAATCTCTAGCAGGCTGCAGGTCGAC

-B-Flap: CAGCAACGCAAGCTTGATGTGGAAAATCTCTAGCA

-A: GGCTGCAGGTCGAC

-B: CAGCAACGCAAGCTTG

-C: GTCGACCTGCAGCCCAAGCTTGCGTTGCTG

## Supporting Information

Figure S1 Cell cycle arrest defects of the *yp30* complementation group. (A) *yp30* mutant worms fail to arrest at the G2 stage following DNA damage. Wild type and *yp30* worms expressing cyclin B1 fused to YFP (gift from Michael O. Hengartner), unirradiated or irradiated (60 Gy) and assayed after 8h. (B) Representative pictures of N2-wild type and gen-1 mitotic germ lines with and without IR treatment. The black arrow depicts a small nucleus of an untreated wild type germ line. The white arrow indicates an enlarged nucleus in an IR treated wild type germ line. Numbers in each panel indicate the respective numbers of nuclei. *yp42*, *yp45*, and *yp30/tm2940* trans-heterozygotes are all defective in IR induced cell cycle arrest. The number of mitotic cells is indicated in each panel.
(3.31 MB TIF)

Figure S2 Quantification and time course analysis of IR dependent cell cycle arrest as described in Figure 1A. Error bars represent s.e.m.
(0.33 MB TIF)

Figure S3 Alignment of GEN-1 N (upper panel) and I domains (lower panel). Alignments were performed as described in Figure 1. A conserved aspartate residue corresponding to amino acid 77 of human XPG is located in the catalytic centre of the N-domain and glutamate 791 and 793 within the I-domain, indicated by arrows. The gap (marked by dots) in the alignment of the I-domain indicates a region with less homology that was removed from the alignment. The alignment of the N-domain and I-domain corresponds to amino acids 1 to 81 and 766 to 863 of human XPG respectively. Ce, *Caenorhabditis elegans*, An, *Aspergillus nidulans*, Bm, *Brugia malayi*, Ca, *Candida albicans*, Ci, *Ciona intestinalis*, Dm, *Drosophila melanogaster*, Gg, *Gallus gallus*, Hs, *Homo sapiens*, Nv, *Nematostella vectensis*, Pp, *Pichia pasteuris*, Sc, *Saccharomyces cerevisiae*, Sp, *Schizosaccharomyces pombe*.
(1.98 MB TIF)

Figure S4 Purification of GEN-1 and in vitro nuclease assays. (A) Purification of recombinant *C. elegans* GEN-1, (pGA532), GEN-1(E135A), (pGA541) and GEN-1(yp30) (pGA543) (left panel) and the human GEN1 amino acid 1-527 fragment (right panel).* indicates an nonspecific band. The arrow indicates GEN-1 while the arrowhead indicates GEN-1(yp30). GEN-1 fragments were cloned into a pGEX derivative containing a C-terminal 6-histidine tag, and induced overnight with 0.5 mM IPTG at 20°C in BL21(DE3) CodonPlus *E.coli* cells and purified on a cobalt column (Talon, Clontech) following the manufacturers instructions. (B) Nuclease assay of human and *C. elegans* GEN-1 on Jbm5 junction substrate. The cleavage assay was performed at 37°C for 30 min. Respective cleavage sites are represented on the right panel. (C) GEN-1 cleaves specifically Holliday Junction structures in vitro. Holliday Junction, 5′ flap, duplex DNA, single-stranded DNA and 3′ overhang were subjected to nuclease assay.
(3.07 MB TIF)

Figure S5 The *gen-1 (tm2940)* deletion but not *gen-1 (yp30)* leads to DNA repair defects. (A), exposure to MMS, (B) UV irradiation, (C) exposure to Nitrogen Mustard, and (D) to exposure to HU. Assays were performed using L4 larvae as described [40]. Error bars represent s.e.m.
(1.12 MB TIF)

Figure S6 RPA-1 loading occurs in *gen-1 (tm2940)*. (A) RPA-1 (green) and RAD-51 foci (red) from wild type and *gen-1 (tm2940)* worms dissected for immunostaining 60 minutes after treatment (30 Gy). Scale bar is 10 µm. Statistical analysis of RPA-1 (B) and RAD-51 foci formation (C). (n = 20 cells), error bars represent s.e.m.. p-values for the comparison between wild type and mutants are between 0.27 and 0.93 indicating that there is no statistically significant difference in RPA-1 foci formation between wild type and the respective mutants.
(3.37 MB TIF)

Figure S7 DNA end joining assays and GEN-1 antibodies purification. (A) *gen-1 (tm2940)* worms are wild type for a DNA end-joining assay affecting somatic cells [59]. Briefly, adult worms are allowed to lay eggs and are removed from the plate where eggs are left for 3 h before being treated with the indicated dose of IR. 48 h later the number of worms that have reached L4 stage worms are counted. (B) Purification of recombinant GEN-1 and antibody generation. (B) Full length GEN-1 fused to an N-terminal His tag (pGA343) was purified using standard procedures and used to immunise one guinea pig. (C) Sera were affinity purified using an N-terminal and C-terminal GEN-1 fragment fused to MBP (pGA346 and pGA348, see Materials and Methods).
(0.67 MB TIF)

Figure S8 Absence of mortal germ line defect in *gen-1* mutants and *gen-1 hpr-17* synthetic lethality. (A) *gen-1 (tm2940)* worms do not have a mortal germ line phenotype indicative of telomere defects. Worm lines were propagated over 30 generations as described [60]. Approximate brood size is indicated. The *trt-1(tm899)* mutant deleting the catalytic subunit of the worm telomerase was used as a positive control. (B) Confirmation of *gen-1 hpr-17* synthetic lethality by double mutant analysis. 10 *gen-1(tm2940); hpr-17(tm1579)*/+F2 lines were selected by PCR, and F3 progeny was scored for the *hpr-17(tm1579)* allele. Out of a total of 36 F3 adult worms 19 were wild type, 17 were heterozygous for *hpr-17(tm1579)* and none was homozygous for *hpr-17(tm1579)*. (C) *gen-1(yp30)* does not enhance the IR hypersensitivity of *hpr-17(tm1579)*. Assays were performed as described in Figure 3.
(0.53 MB TIF)

Figure S9 Increased level of RAD51 foci in *mrt-2(e2663) gen-1(RNAi)* premeiotic germ lines. Foci were quantified 24 h post L4 (right panel). A projection of five z-stacks is shown using SoftWorXs Applied Precision 3.1 for clarity. For quantification the entire germline was projected and foci were counted (n = 5). Error bars represent s.e.m.
(2.84 MB TIF)

Figure S10 Analysis of *gen-1* and *gen-1 atm-1* double mutants by the L4 IR survival assay. Error bars represent s.e.m.
(0.49 MB TIF)
