# Supplementary figures and images for: The Caenorhabditis elegans Homolog of Gen1/Yen1 Resolvases Links DNA Damage Signaling to DNA Double-Strand Break Repair
Source: PLoS Genet. 2010 Jul 15;6(7):e1001025. doi: 10.1371/journal.pgen.1001025 (PMC2908289; doi:10.1371/journal.pgen.1001025)

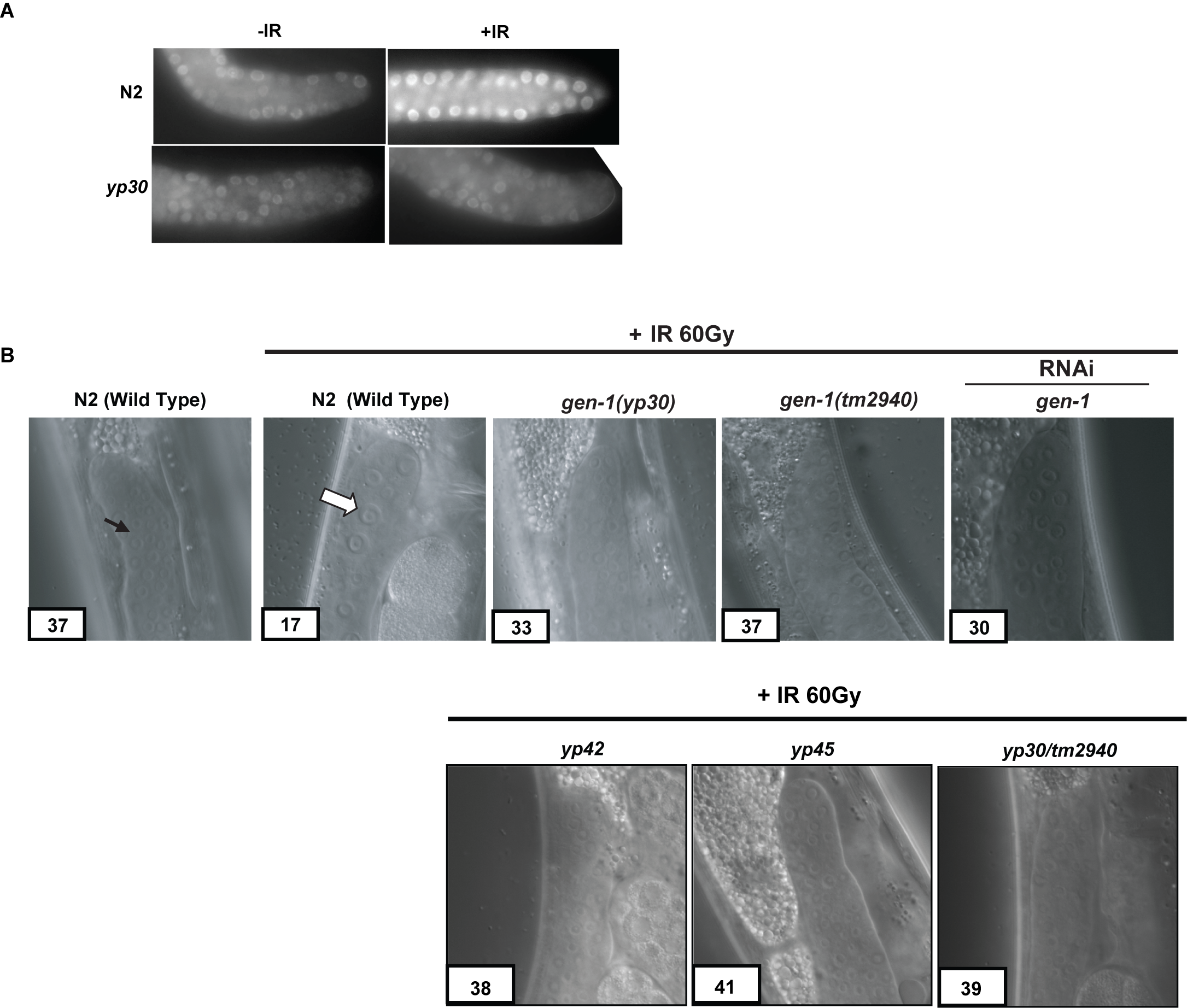

Supplement: Figure S1 — Cell cycle arrest defects of the yp30 complementation group. (A) yp30 mutant worms fail to arrest at the G2 stage following DNA damage. Wild type and yp30 worms expressing cyclin B1 fused to YFP (gift from Michael O. Hengartner), unirradiated or irradiated (60 Gy) and assayed after 8h. (B) Representative pictures of N2-wild type and gen-1 mitotic germ lines with and without IR treatment. The black arrow depicts a small nucleus of an untreated wild type germ line. The white arrow indicates an enlarged nucleus in an IR treated wild type germ line. Numbers in each panel indicate the respective numbers of nuclei. yp42, yp45, and yp30/tm2940 trans-heterozygotes are all defective in IR induced cell cycle arrest. The number of mitotic cells is indicated in each panel. (3.31 MB TIF) [file pgen.1001025.s001.tif]

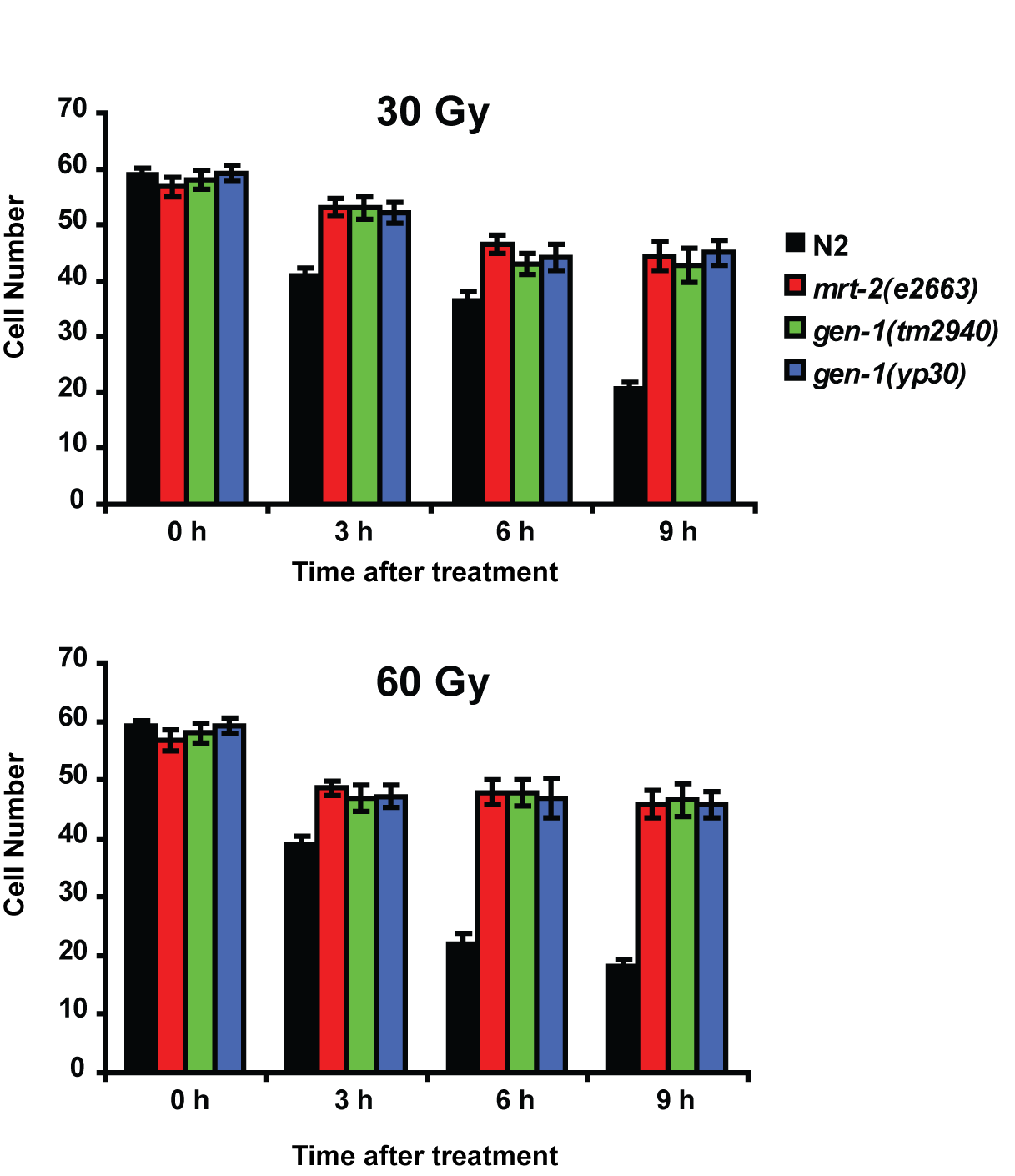

Supplement: Figure S2 — Quantification and time course analysis of IR dependent cell cycle arrest as described in Figure 1A. Error bars represent s.e.m. (0.33 MB TIF) [file pgen.1001025.s002.tif]

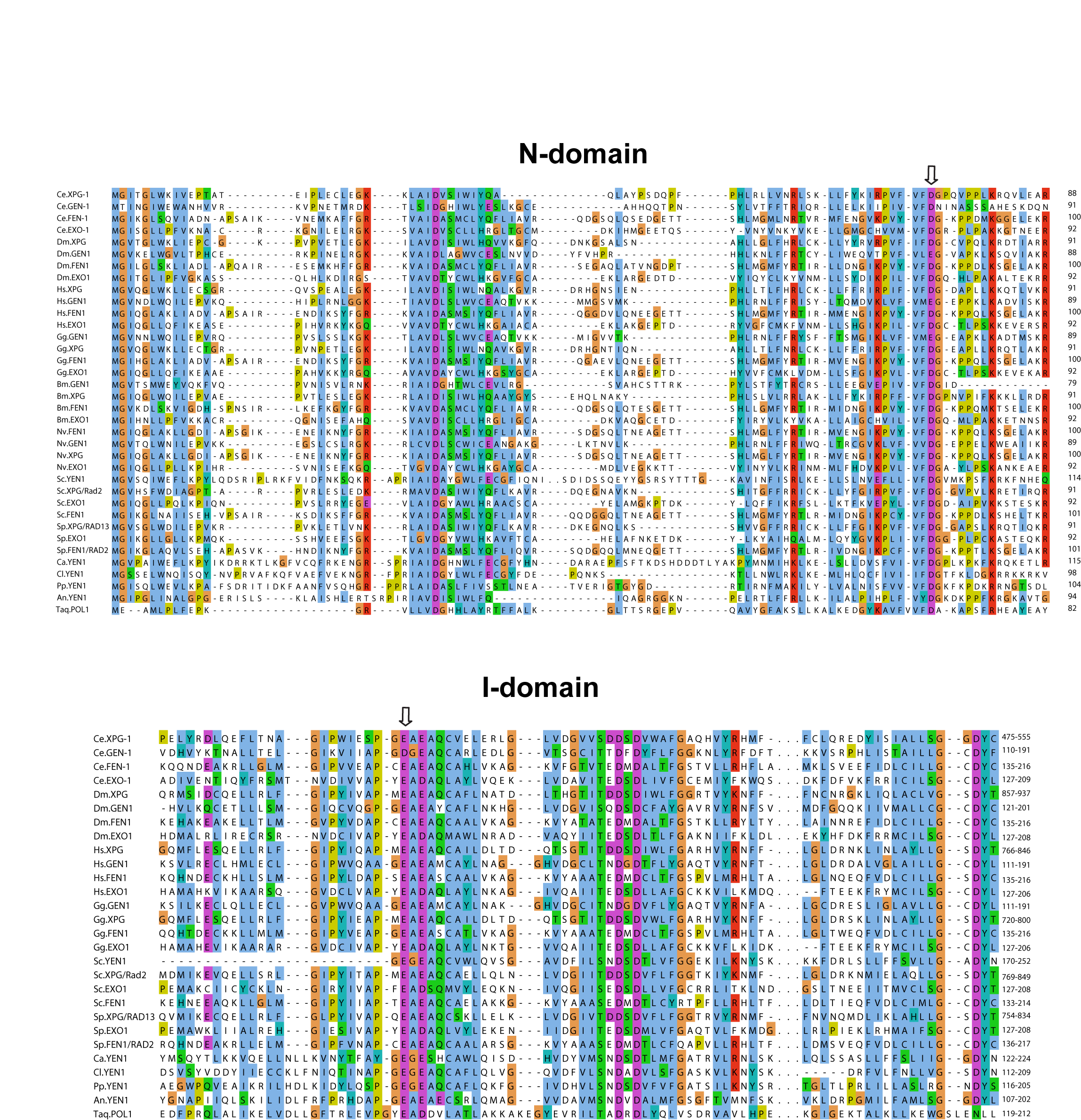

Supplement: Figure S3 — Alignment of GEN-1 N (upper panel) and I domains (lower panel). Alignments were performed as described in Figure 1. A conserved aspartate residue corresponding to amino acid 77 of human XPG is located in the catalytic centre of the N-domain and glutamate 791 and 793 within the I-domain, indicated by arrows. The gap (marked by dots) in the alignment of the I-domain indicates a region with less homology that was removed from the alignment. The alignment of the N-domain and I-domain corresponds to amino acids 1 to 81 and 766 to 863 of human XPG respectively. Ce, Caenorhabditis elegans, An, Aspergillus nidulans, Bm, Brugia malayi, Ca, Candida albicans, Ci, Ciona intestinalis, Dm, Drosophila melanogaster, Gg, Gallus gallus, Hs, Homo sapiens, Nv, Nematostella vectensis, Pp, Pichia pasteuris, Sc, Saccharomyces cerevisiae, Sp, Schizosaccharomyces pombe. (1.98 MB TIF) [file pgen.1001025.s003.tif]

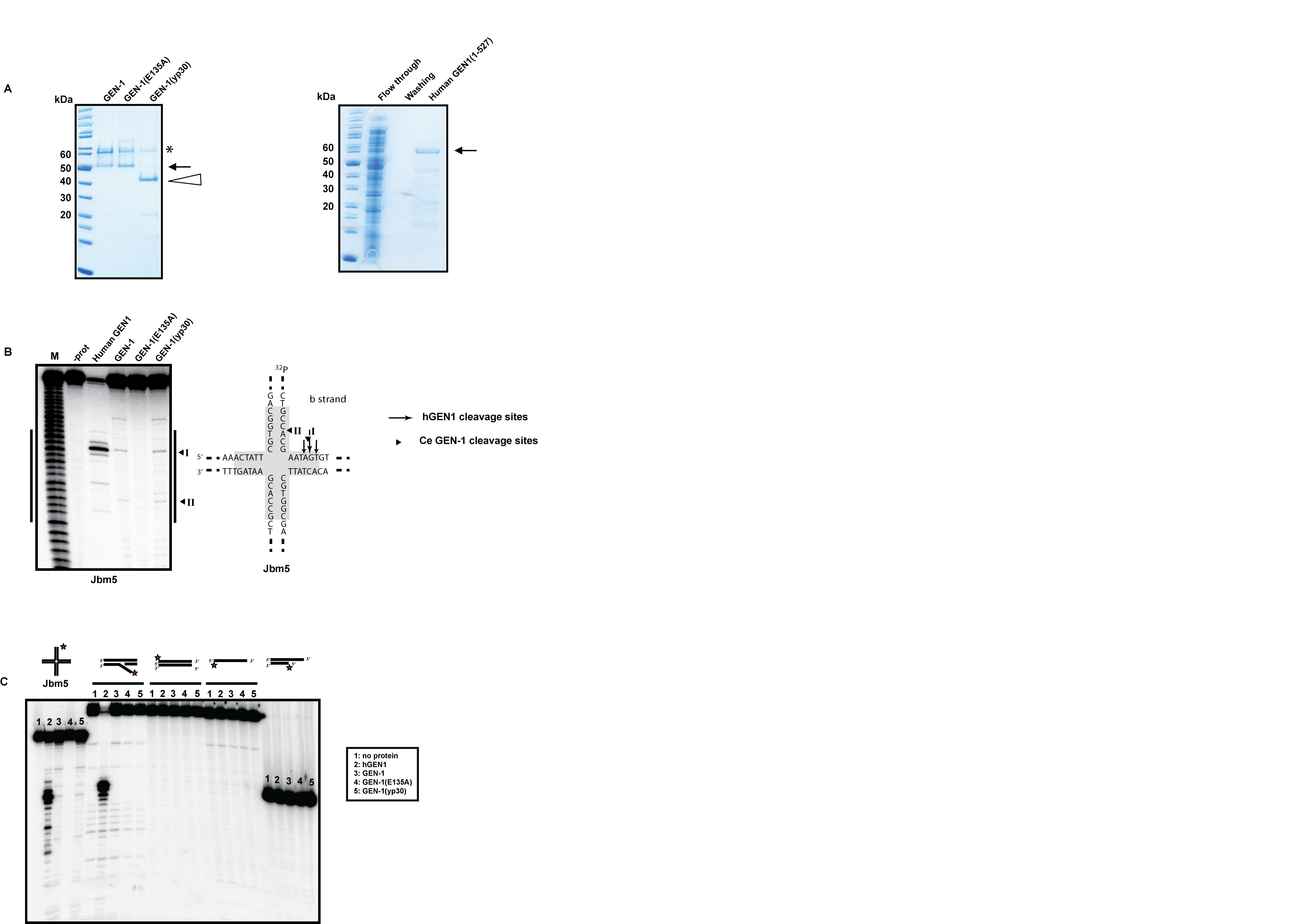

Supplement: Figure S4 — Purification of GEN-1 and in vitro nuclease assays. (A) Purification of recombinant C. elegans GEN-1, (pGA532), GEN-1(E135A), (pGA541) and GEN-1(yp30) (pGA543) (left panel) and the human GEN1 amino acid 1-527 fragment (right panel).* indicates an nonspecific band. The arrow indicates GEN-1 while the arrowhead indicates GEN-1(yp30). GEN-1 fragments were cloned into a pGEX derivative containing a C-terminal 6-histidine tag, and induced overnight with 0.5 mM IPTG at 20°C in BL21(DE3) CodonPlus E.coli cells and purified on a cobalt column (Talon, Clontech) following the manufacturers instructions. (B) Nuclease assay of human and C. elegans GEN-1 on Jbm5 junction substrate. The cleavage assay was performed at 37°C for 30 min. Respective cleavage sites are represented on the right panel. (C) GEN-1 cleaves specifically Holliday Junction structures in vitro. Holliday Junction, 5′ flap, duplex DNA, single-stranded DNA and 3′ overhang were subjected to nuclease assay. (3.07 MB TIF) [file pgen.1001025.s004.tif]

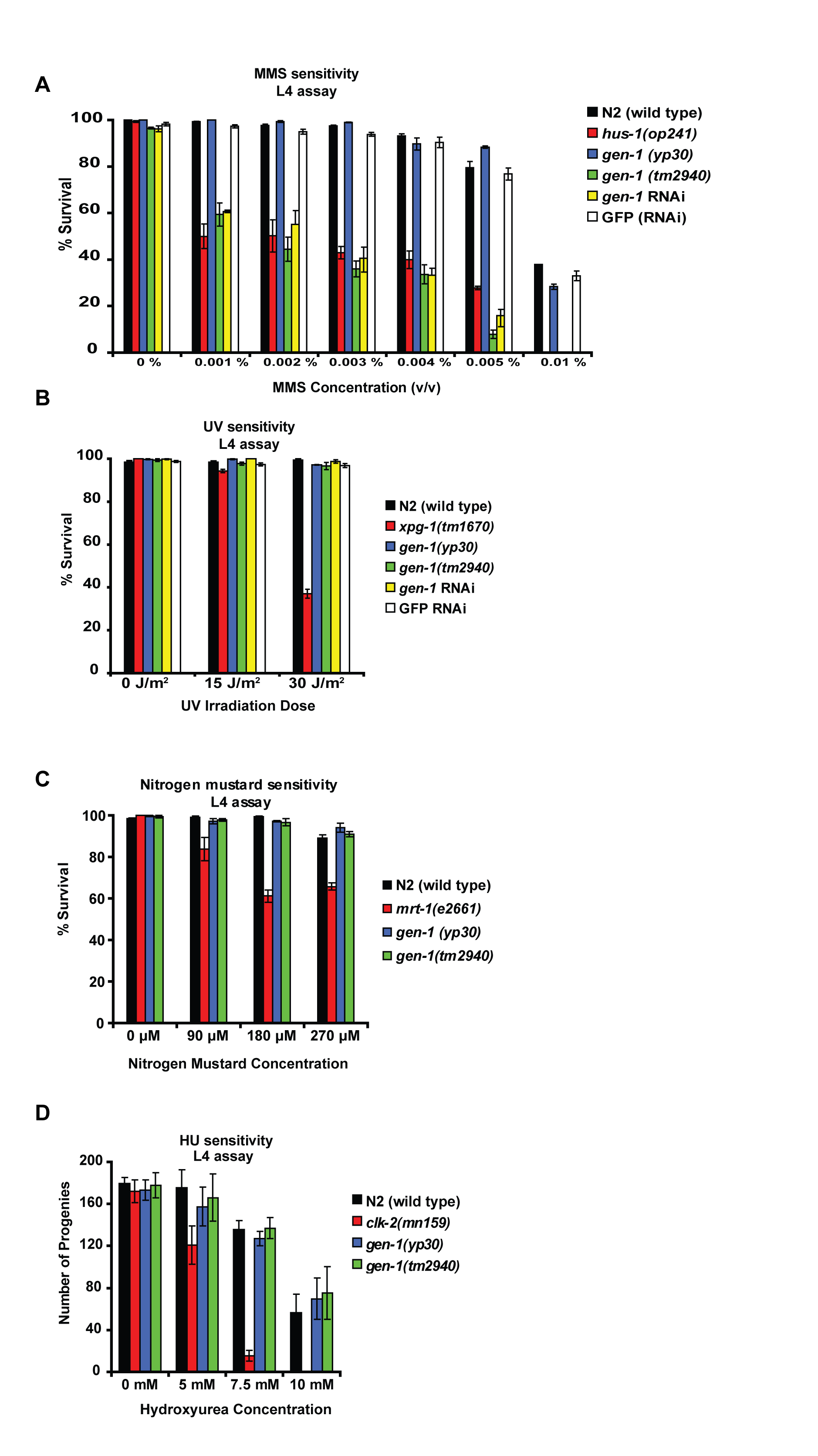

Supplement: Figure S5 — The gen-1 (tm2940) deletion but not gen-1 (yp30) leads to DNA repair defects. (A), exposure to MMS, (B) UV irradiation, (C) exposure to Nitrogen Mustard, and (D) to exposure to HU. Assays were performed using L4 larvae as described [40]. Error bars represent s.e.m. (1.12 MB TIF) [file pgen.1001025.s005.tif]

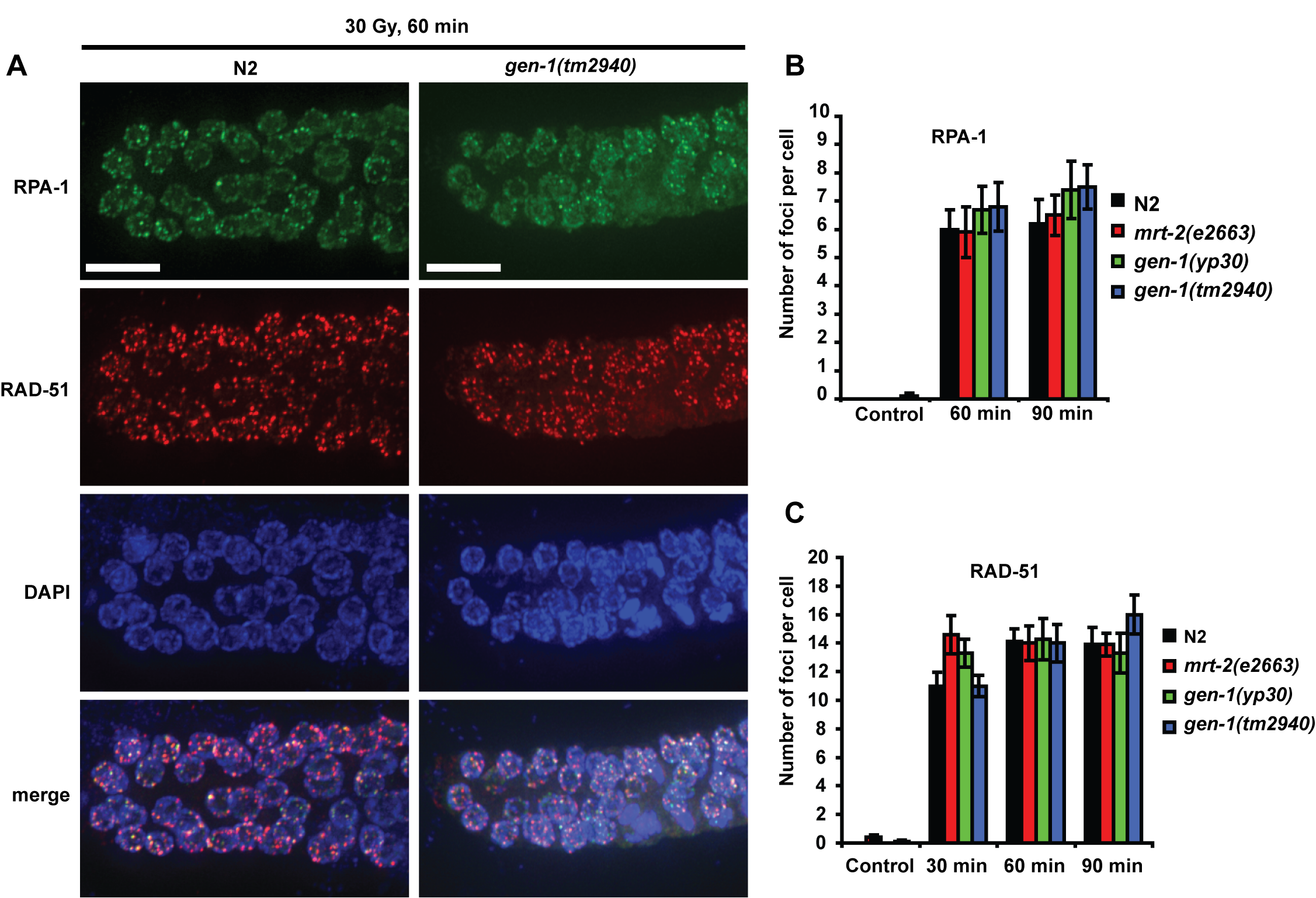

Supplement: Figure S6 — RPA-1 loading occurs in gen-1 (tm2940). (A) RPA-1 (green) and RAD-51 foci (red) from wild type and gen-1 (tm2940) worms dissected for immunostaining 60 minutes after treatment (30 Gy). Scale bar is 10 µm. Statistical analysis of RPA-1 (B) and RAD-51 foci formation (C). (n = 20 cells), error bars represent s.e.m.. p-values for the comparison between wild type and mutants are between 0.27 and 0.93 indicating that there is no statistically significant difference in RPA-1 foci formation between wild type and the respective mutants. (3.37 MB TIF) [file pgen.1001025.s006.tif]

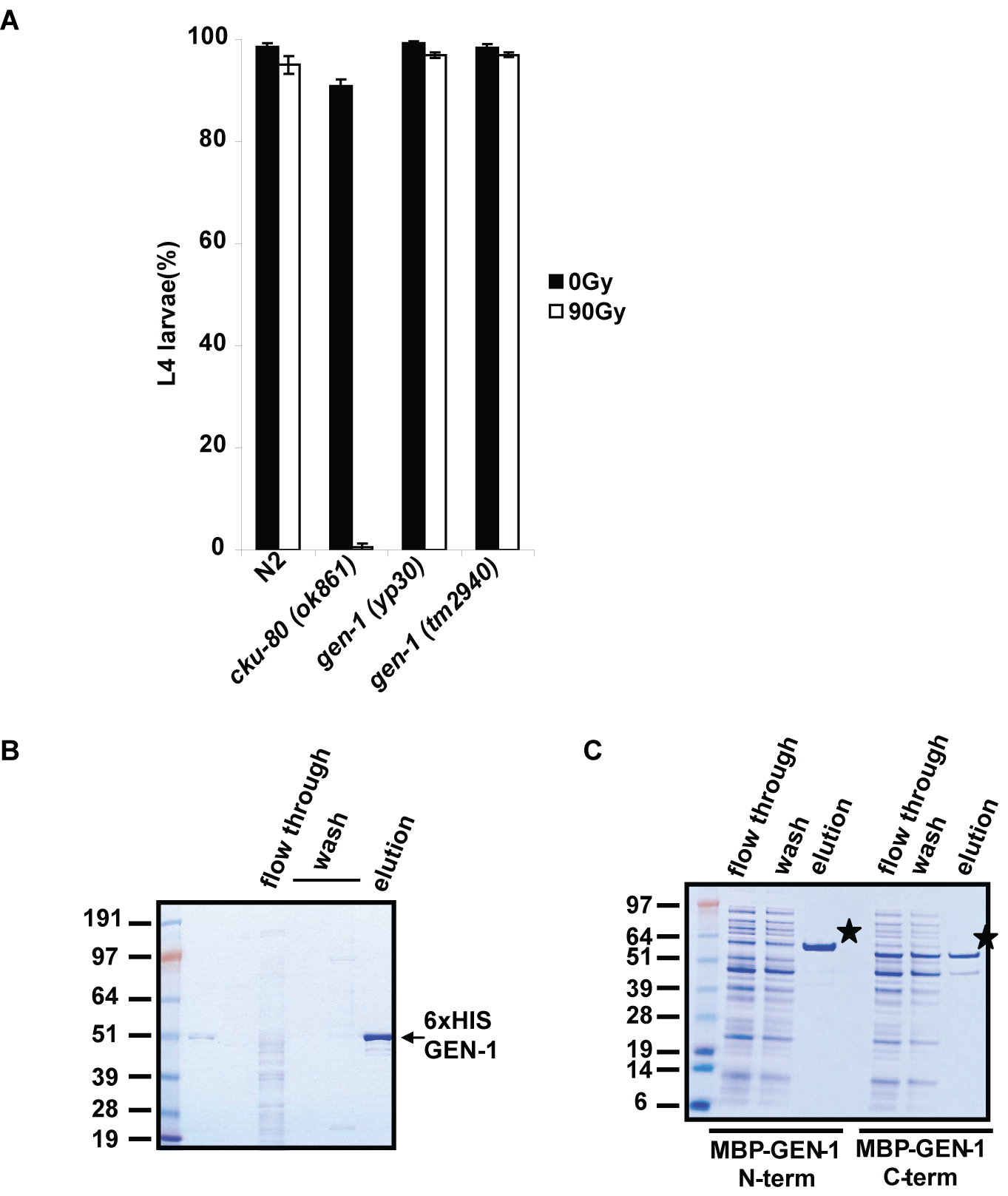

Supplement: Figure S7 — DNA end joining assays and GEN-1 antibodies purification. (A) gen-1 (tm2940) worms are wild type for a DNA end-joining assay affecting somatic cells [59]. Briefly, adult worms are allowed to lay eggs and are removed from the plate where eggs are left for 3 h before being treated with the indicated dose of IR. 48 h later the number of worms that have reached L4 stage worms are counted. (B) Purification of recombinant GEN-1 and antibody generation. (B) Full length GEN-1 fused to an N-terminal His tag (pGA343) was purified using standard procedures and used to immunise one guinea pig. (C) Sera were affinity purified using an N-terminal and C-terminal GEN-1 fragment fused to MBP (pGA346 and pGA348, see Materials and Methods). (0.67 MB TIF) [file pgen.1001025.s007.tif]

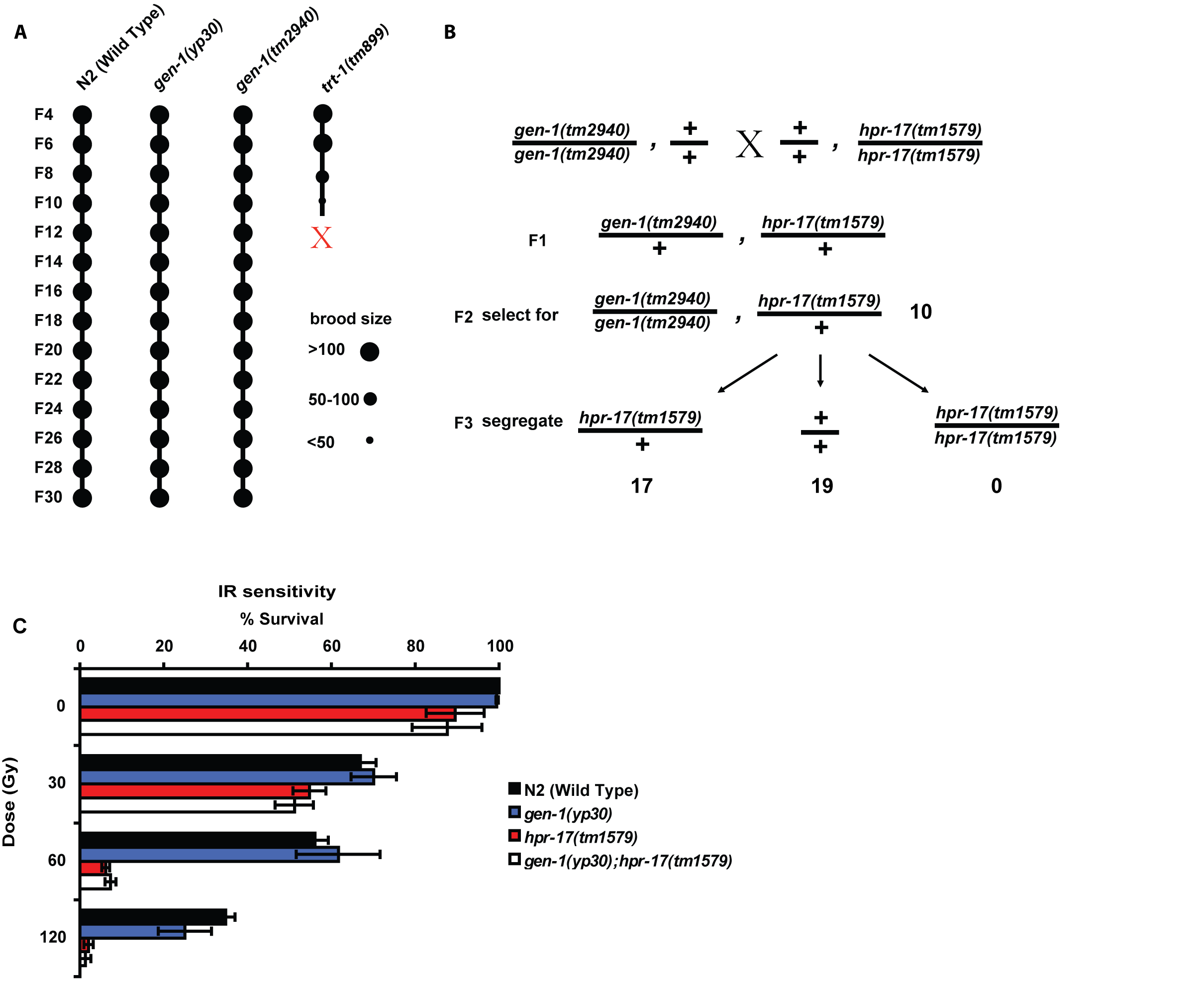

Supplement: Figure S8 — Absence of mortal germ line defect in gen-1 mutants and gen-1 hpr-17 synthetic lethality. (A) gen-1 (tm2940) worms do not have a mortal germ line phenotype indicative of telomere defects. Worm lines were propagated over 30 generations as described [60]. Approximate brood size is indicated. The trt-1(tm899) mutant deleting the catalytic subunit of the worm telomerase was used as a positive control. (B) Confirmation of gen-1 hpr-17 synthetic lethality by double mutant analysis. 10 gen-1(tm2940); hpr-17(tm1579)/+F2 lines were selected by PCR, and F3 progeny was scored for the hpr-17(tm1579) allele. Out of a total of 36 F3 adult worms 19 were wild type, 17 were heterozygous for hpr-17(tm1579) and none was homozygous for hpr-17(tm1579). (C) gen-1(yp30) does not enhance the IR hypersensitivity of hpr-17(tm1579). Assays were performed as described in Figure 3. (0.53 MB TIF) [file pgen.1001025.s008.tif]

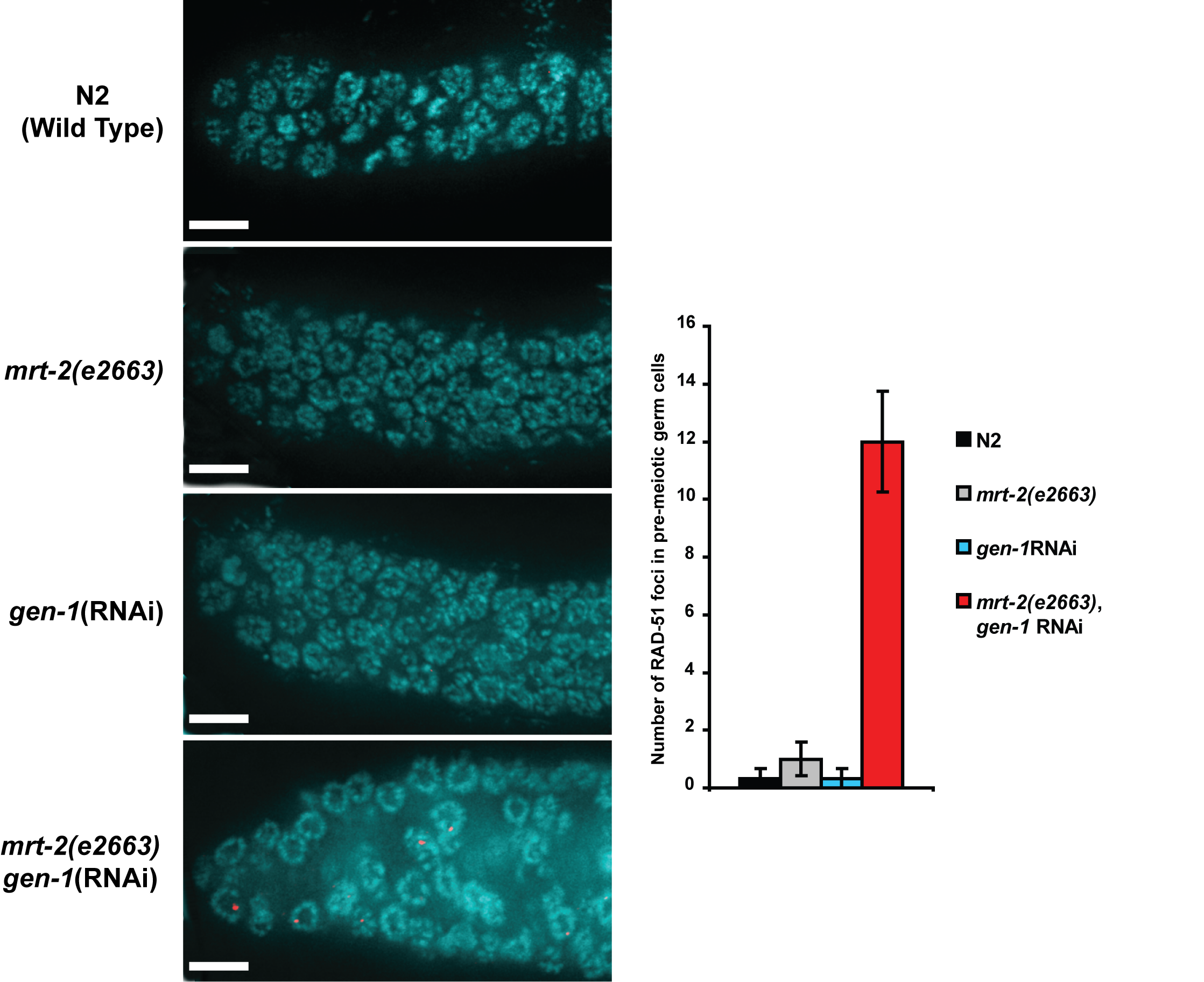

Supplement: Figure S9 — Increased level of RAD51 foci in mrt-2(e2663) gen-1(RNAi) premeiotic germ lines. Foci were quantified 24 h post L4 (right panel). A projection of five z-stacks is shown using SoftWorXs Applied Precision 3.1 for clarity. For quantification the entire germline was projected and foci were counted (n = 5). Error bars represent s.e.m. (2.84 MB TIF) [file pgen.1001025.s009.tif]

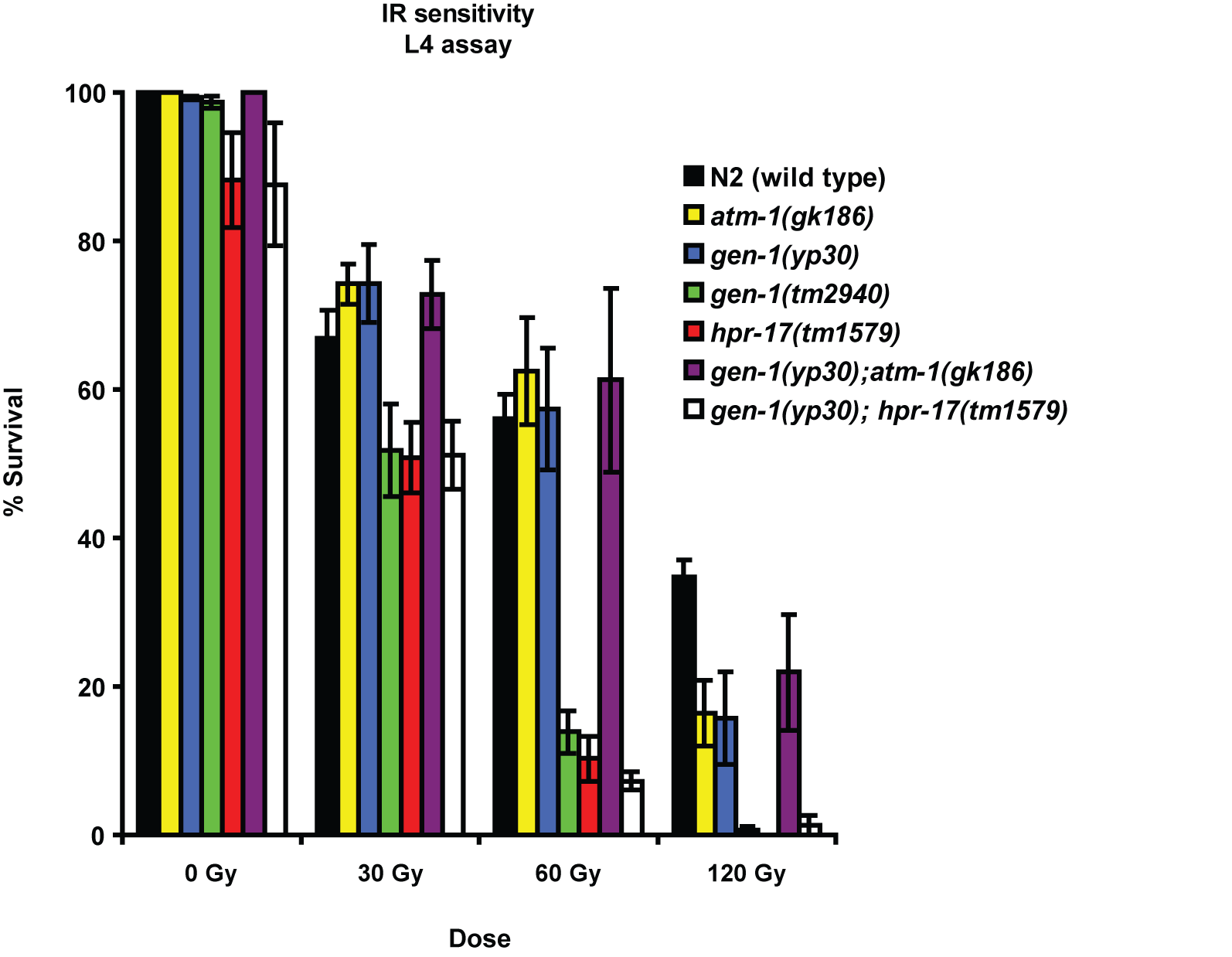

Supplement: Figure S10 — Analysis of gen-1 and gen-1 atm-1 double mutants by the L4 IR survival assay. Error bars represent s.e.m. (0.49 MB TIF) [file pgen.1001025.s010.tif]
